# Supplementary material for: Quality assurance practices in tuberculosis diagnostic health facilities in Ethiopia
Source: PLoS One. 2022 Jun 9;17(6):e0269601. doi: 10.1371/journal.pone.0269601 (PMC9182608; doi:10.1371/journal.pone.0269601)
Supplement: Attachment — Submitted filename: PONE-D-21-20924_reviewed by HS.pdf [file pone.0269601.s001.pdf]

PLOS ONE

Quality assurance practices in tuberculosis diagnostic health facilities in Ethiopia

--Manuscript Draft--

Manuscript Number:	PONE-D-21-20924
Article Type:	Research Article
Full Title:	Quality assurance practices in tuberculosis diagnostic health facilities in Ethiopia
Short Title:	Quality assurance practices in tuberculosis diagnostic health facilities in Ethiopia
Corresponding Author:	Yeshiwork Abebaw<br>Ethiopian Public Health Institute<br>Gulele, AA ETHIOPIA
Keywords:	Keywords: Quality Assurance Practice, Quality Control, External Quality Assessment
Abstract:	Abstract<br><br>Introduction: The quality of tuberculosis laboratory services in health facilities is a mandatory component of detecting active pulmonary TB cases and treatment follow-up. However, ensuring the quality of laboratory test results is a concern. This study was aimed to assess the quality assurance practices in the tuberculosis diagnostic health facilities of Ethiopia.<br>Method: A cross-sectional study was conducted from October 2018 to March 2019 at nine governmental TB-culture laboratories and 34 randomly selected GeneXpert® MTB/RIF (Xpert® MTB/RIF) testing health facilities in Ethiopia. We went over last year's records and spoke with them about the laboratory's focus. Prior to the data collection, training was given to the data collectors. Descriptive statistics were used to produce results and were presented with tables and graphs.<br>Result: From a total of 34 Xpert® MTB/RIF testing laboratories, 17/34 (50%) of them run Internal quality control (IQC) for Acid-Fast Bacillus (AFB) Microscopy and 23/34 (67.6%) of them had lot-to-lot verification of staining reagents. For the Xpert® MTB/RIF assay, a lot-to-lot verification of cartridge and method validation was performed only in 3/34 (8.8%) and 7/34 (20.6%) of Xpert® MTB/RIF testing laboratories respectively. All TB-culture laboratories were included in the study to run start and end IQC (negative control) during TB-culture sample processing and they performed lot-to-lot verification for Mycobacteria growth Indicator Tube (MGIT) in 8/9 (88.9%) of the TB-culture laboratories. External Quality Assessment (EQA) Proficiency Testing (PT) for AFB microscopy is practiced in 27/34 (79.4%) of Xpert® MTB/RIF testing laboratories and 34 (100.0%) for the Xpert® MTB/RIF assay. TB-Culture PT participation practice among TB-culture laboratories was 8/9 (88.9%). A major challenge for health facilities during PT participation was the AFB PT-sample transportation delay of 11/27 (40.7%) and the Xpert® MTB/RIF assay EQA-PT feedback missing 13/34 (38.2%).<br>Conclusions: This assessment reveals lot-to-lot verification and method validation were not well-practiced. The majority of TB diagnostic health facility laboratories had EQA-PT participation practice, but a significant gap in PT-sample transportation and feedback was identified.
Order of Authors:	Yeshiwork Abebaw
	Abebaw Kebede
	Kirubel Eshetu
	Ephrem Tesfaye
	Mengistu Tadesse
	Waganeh Sinshaw
	Misikir Amare
	Dinka Fikadu Gamtesa
	Betselot Zerihun
	Melak Getu

Powered by Editorial Manager® and ProduXion Manager® from Aries Systems Corporation

	Getachew Sied
	Anteneh Yalew
	Getu Diriba
**Additional Information:**	
**Question**	**Response**
**Financial Disclosure**<br><br>Enter a financial disclosure statement that describes the sources of funding for the work included in this submission. Review the submission guidelines for detailed requirements. View published research articles from *PLOS ONE* for specific examples.<br><br>This statement is required for submission and **will appear in the published article** if the submission is accepted. Please make sure it is accurate.<br><br>**Unfunded studies**<br>Enter: *The author(s) received no specific funding for this work.*<br><br>**Funded studies**<br>Enter a statement with the following details:<br>• Initials of the authors who received each award<br>• Grant numbers awarded to each author<br>• The full name of each funder<br>• URL of each funder website<br>• Did the sponsors or funders play any role in the study design, data collection and analysis, decision to publish, or preparation of the manuscript?<br>• **NO** - Include this sentence at the end of your statement: *The funders had no role in study design, data collection and analysis, decision to publish, or preparation of the manuscript.*<br>• **YES** - Specify the role(s) played.<br><br>* typeset	The author(s) received no specific funding for this work.
**Competing Interests**<br><br>Use the instructions below to enter a competing interest statement for this submission. On behalf of all authors, disclose any competing interests that could be perceived to bias this	The authors have declared that no competing interests exist.

work—acknowledging all financial support and any other relevant financial or non-financial competing interests.<br><br>This statement is required for submission and **will appear in the published article** if the submission is accepted. Please make sure it is accurate and that any funding sources listed in your Funding Information later in the submission form are also declared in your Financial Disclosure statement.<br><br>View published research articles from *PLOS ONE* for specific examples.<br><br>**NO authors have competing interests**<br><br>Enter: *The authors have declared that no competing interests exist.*<br><br>**Authors with competing interests**<br><br>Enter competing interest details beginning with this statement:<br><br>*I have read the journal's policy and the authors of this manuscript have the following competing interests: [insert competing interests here]*<br><br>* typeset	
**Ethics Statement**<br><br>Enter an ethics statement for this submission. This statement is required if the study involved:<br><br>• Human participants<br>• Human specimens or tissue<br>• Vertebrate animals or cephalopods<br>• Vertebrate embryos or tissues<br>• Field research<br><br>Write "N/A" if the submission does not require an ethics statement.	N/A

General guidance is provided below. Consult the submission guidelines for detailed instructions. **Make sure that all information entered here is included in the Methods section of the manuscript.**<br><br>**Format for specific study types**<br><br>**Human Subject Research (involving human participants and/or tissue)**<br>• Give the name of the institutional review board or ethics committee that approved the study<br>• Include the approval number and/or a statement indicating approval of this research<br>• Indicate the form of consent obtained (written/oral) or the reason that consent was not obtained (e.g. the data were analyzed anonymously)<br><br>**Animal Research (involving vertebrate animals, embryos or tissues)**<br>• Provide the name of the Institutional Animal Care and Use Committee (IACUC) or other relevant ethics board that reviewed the study protocol, and indicate whether they approved this research or granted a formal waiver of ethical approval<br>• Include an approval number if one was obtained<br>• If the study involved *non-human primates*, add *additional details* about animal welfare and steps taken to ameliorate suffering<br>• If anesthesia, euthanasia, or any kind of animal sacrifice is part of the study, include briefly which substances and/or methods were applied<br><br>**Field Research**<br><br>Include the following details if this study involves the collection of plant, animal, or other materials from a natural setting:<br>• Field permit number<br>• Name of the institution or relevant body that granted permission	
**Data Availability**<br><br>Authors are required to make all data underlying the findings described fully	Yes - all data are fully available without restriction

available, without restriction, and from the time of publication. PLOS allows rare exceptions to address legal and ethical concerns. See the PLOS Data Policy and FAQ for detailed information.<br><br>A Data Availability Statement describing where the data can be found is required at submission. Your answers to this question constitute the Data Availability Statement and **will be published in the article**, if accepted.<br><br>**Important:** Stating 'data available on request from the author' is not sufficient. If your data are only available upon request, select 'No' for the first question and explain your exceptional situation in the text box.<br><br>Do the authors confirm that all data underlying the findings described in their manuscript are fully available without restriction?	
**Describe where the data may be found in full sentences. If you are copying our sample text, replace any instances of XXX with the appropriate details.**<br><br>• If the data are **held or will be held in a public repository**, include URLs, accession numbers or DOIs. If this information will only be available after acceptance, indicate this by ticking the box below. For example: *All XXX files are available from the XXX database (accession number(s) XXX, XXX.*).<br>• If the data are all contained **within the manuscript and/or Supporting Information files**, enter the following: *All relevant data are within the manuscript and its Supporting Information files.*<br>• If neither of these applies but you are able to provide **details of access elsewhere**, with or without limitations, please do so. For example:<br><br>*Data cannot be shared publicly because of [XXX]. Data are available from the XXX Institutional Data Access / Ethics*	All relevant data are within the manuscript.

Committee (contact via XXX) for researchers who meet the criteria for access to confidential data.<br><br>*The data underlying the results presented in the study are available from (include the name of the third party and contact information or URL).*<br>• This text is appropriate if the data are owned by a third party and authors do not have permission to share the data.<br><br>* typeset	
Additional data availability information:	

Powered by Editorial Manager® and ProduXion Manager® from Aries Systems Corporation

Quality assurance practices in tuberculosis diagnostic health facilities in Ethiopia

Yeshiwork Abebaw[1,2*], Abebaw Kebede[1], Kirubel Eshetu[1], Ephrem Tesfaye[1], Mengestu Tadesse[1], Waganeh Sinshaw[1,2], Misiker Amare[1], Dinka Fikadu[1], Betselot Zerihun[1,2], Melak Getu[1,2], Getachew Seid[1], Anteneh Yalew[1,,3,4], Getu Diriba[1]

[1]Ethiopian Public Health Institute, Addis Ababa, Ethiopia

[2]Department of Microbiology, Immunology and Parasitology College of Health Sciences, Addis Ababa University, Addis Ababa, Ethiopia

[3]Division of Epidemiology and Biostatistics, Department of Global Health, Faculty of Medicine and Health Sciences, Stellenbosch University, Cape Town, South Africa

[4]Department of Statistics, College of Natural and Computational Sciences, Addis Ababa University, Addis Ababa, Ethiopia, email: anteneh2123@gmail.com

[1]Yeshiwork Abebaw Ethiopian Public Health Institute, email: Yeshi885@gmail.com, Addis Ababa, Ethiopia, Corresponding Author

[1]Abebaw Kebede, Ethiopian Public Health Institute, email: jkabebaw@gmail.com, Addis Ababa, Ethiopia.

[1]Kirubel Eshetu, Ethiopian Public Health Institute, email: kirub1625@gmail.com, Addis Ababa, Ethiopia

[1]Ephrem Tesfaye[1], Ethiopian public health institute, email: ephremt13@gmail.com Addis Ababa, Ethiopia

[1]Mengistu Tadesse, Ethiopian Public Health Institute, email: mengistutadesse86@gmail.com, Addis Ababa, Ethiopia

[1]Waganeh Sinshaw Ethiopian public health institute, email: wagamels@gmail.com Addis Ababa, Ethiopia

[1]Misikir Amare, Ethiopian Public Health Institute, email: misikiramare.ma@gmail.com, Addis Ababa, Ethiopia

[1]Dinka Fikadu Gamtesa, Ethiopian Public Health Institute, email: ejeta430@gmail.com, Addis Ababa, Ethiopia

[1]Betselot Zerihun, Ethiopian public health institute, email: betselotzerihun3@gmail.com, Addis Ababa, Ethiopia

[1]Melak Getu, Ethiopian public health institute, email: melakgetu2005@gmail.com, Addis Ababa, Ethiopia

[1]Getachew Sied, Ethiopian public health institute, email: gech1365@gmail.com, Addis Ababa, Ethiopia

[1]Anteneh Yalew, Ethiopian public health institute, email: anteneh2123@gmail.com, Addis Ababa, Ethiopia

[1]Getu Diriba, Ethiopian Public Health Institute, email:getud2020@gmail.com, Addis Ababa, Ethiopia

[*]Corresponding author Yeshiwork Abebaw

Ethiopian Public Health Institute

Phone: +251913447415. Fax: +2510112780431

P.O.Box 1242, Addis Ababa, Ethiopia

Email: Yeshi885@gmail.com

Abstract

Introduction: The quality of tuberculosis laboratory services in health facilities is a mandatory component of detecting active pulmonary TB cases and treatment follow-up. However, ensuring the quality of laboratory test results is a concern. This study was aimed to assess the quality assurance practices in the tuberculosis diagnostic health facilities of Ethiopia.

Method: A cross-sectional study was conducted from October 2018 to March 2019 at nine governmental TB-culture laboratories and 34 randomly selected GeneXpert® MTB/RIF (Xpert® MTB/RIF) testing health facilities in Ethiopia. We went over last year's records and spoke with them about the laboratory's focus. Prior to the data collection, training was given to the data collectors. Descriptive statistics were used to produce results and were presented with tables and graphs.

Result: From a total of 34 Xpert® MTB/RIF testing laboratories, 17/34 (50%) of them run Internal quality control (IQC) for Acid-Fast Bacillus (AFB) Microscopy and 23/34 (67.6%) of them had lot-to-lot verification of staining reagents. For the Xpert® MTB/RIF assay, a lot-to-lot verification of cartridge and method validation was performed only in 3/34 (8.8%) and 7/34 (20.6%) of Xpert® MTB/RIF testing laboratories respectively. All TB-culture laboratories were included in the study to run start and end IQC (negative control) during TB-culture sample processing and they performed lot-to-lot verification for Mycobacteria growth Indicator Tube (MGIT) in 8/9 (88.9%) of the TB-culture laboratories. External Quality Assessment (EQA) Proficiency Testing (PT) for AFB microscopy is practiced in 27/34 (79.4%) of Xpert® MTB/RIF testing laboratories and 34 (100.0%) for the Xpert® MTB/RIF assay. TB-Culture PT participation practice among TB-culture laboratories was 8/9 (88.9%). A major challenge for health facilities during PT participation was the AFB PT-sample transportation delay of 11/27 (40.7%) and the Xpert® MTB/RIF assay EQA-PT feedback missing 13/34 (38.2%).

Conclusions: This assessment reveals lot-to-lot verification and method validation were not well-practiced. The majority of TB diagnostic health facility laboratories had EQA-PT participation practice, but a significant gap in PT-sample transportation and feedback was identified.

Keywords: Quality Assurance Practice, Quality Control, External Quality Assessment

Introduction

The tuberculosis (TB) laboratory plays an essential role in diagnosing TB and monitoring its treatment (1). In the last decade, TB screening methods have been improved beyond smear microscopy ~~and~~ TB culture and drug susceptibility testing (DST) using solid media (1). Innovations in TB diagnostics (near point-of-care testing) have ensured accessibility and affordability in low and middle-income countries (1,2). With the expansion of TB testing, quality assurance of the test remains an important element that should be addressed in the TB control effort (3).

The quality assurance of testing systematically monitors routine technical laboratory activities, mainly ensured through quality control (QC), external quality assessment (EQA), and continuous quality improvement (3,4). Quality control ensures the TB diagnostic laboratory controls operations, including equipment checks and checking new lots of staining solutions (5,6).

EQA plays a vital role in the monitoring of laboratory performance and maintaining laboratory quality service (7,8). It also allows participating laboratories to assess their capabilities by comparing their results with those in other laboratories in the network (7,9). One of the end TB strategic goals in 2020 is to enroll all TB diagnostic facilities with EQA (10). EQA for tuberculosis diagnosis and drug resistance is practiced in different countries with proficiency testing and subsequent technical assistance to improve the DST quality of participating laboratories (11,12).

Strengthening Laboratory Management towards Accreditation (SLMTA) is a training framework that WHO has initiated to strengthen public health laboratory quality through its regional office. Ethiopia joined the SLMTA program for the first time in 2010, using selected health facilities (13). Later, TB-SLMTA was introduced in TB culture and DST laboratories in 2013. Since then, the Ethiopia National TB prevention and control program with partners has been working to expand the customized quality management system all over the country's TB diagnostic facilities (14). Subsequently, regional health institutes or sub-regional laboratories, or designated EQA centers have been conducting onsite supervision and blinded rechecking of TB diagnostic centers in their respective catchment areas in accordance with national EQA guidelines (15). The National TB Reference Laboratory (NRL) of Ethiopian Public Health Institute (EPHI) provides PT for the Xpert® MTB/RIF assay, TB culture and identification, and LPA first and second-line DST for TB diagnostic facilities. Additionally, One-World Accuracy, an international PT provider, supplies PT for selected TB diagnostic health facilities. Also CDC-Atlanta offers PT for selected Xpert® MTB/RIF testing sites.

There are national and international recommendations on how often QC and EQA should be performed for each TB testing method (16,17). However, for a variety of reasons, many health facilities lack the implementation of QC and participation in EQA by diagnostic facilities (18). In Ethiopia, some studies have been conducted evaluating the QA practices of TB diagnostic centers (8,19). However, these studies did not cover TB culture and Xpert® MTB/RIF testing laboratories, while the country currently recommends the Xpert® MTB/RIF assay for rapid detection of TB and TB culture for MDR-TB treatment monitoring. Therefore, this study aimed to assess the QA practices of TB culture and Xpert® MTB/RIF testing laboratories in Ethiopia.

Materials and Methods

Study design and setting

A cross-sectional study was conducted in 34 selected government Xpert® MTB/RIF testing laboratories and nine TB culture laboratories from October 2018 to March 2019. Ethiopia is the second-populated country in Africa, with an estimated 112.0 million people in 2019 (20). In the country, there are ten TB and DST culture laboratories. During the study period, there were 170 Xpert® MTB/RIF testing health facilities, while recently the machines were distributed to more than 300 TB diagnostic health facilities. The NTRL of EPHI is a national reference center and has been the sole PT provider for molecular testing (Xpert® MTB/RIF assay, Genotype MTBDRplus assay, and Genotype MTBDRsl assay) and PT for TB culture in Ethiopia.

Sampling Techniques and Sample Size

Stratified random sampling was used. The sample size was determined for facility-based assessment surveys by computing the following formula (21):

$$n = \left[\left[\left(\frac{z^2_{\alpha/2} pq + ME^2}{ME^2 + \frac{z^2_{\alpha/2} pq}{N}} \right) \right] * d \right]$$

Where n is the sample size to be determined, the value of Z for 95% power is 1.96, p =50%, and d is the margin of allowable error (0.15). Therefore, based on the above formula, all (nine) governmental TB-culture and 34 Xpert® MTB/RIF assay testing health facilities for Xpert® MTB/RIF were included in the study.

Inclusion criteria

All governmental TB-culture and Xpert® MTB/RIF assay testing health facilities with at least one year of health-care experience in the area.

Data Collection Methods

The study assessed the main QA practices in selected TB diagnostic centers with TB culture and Xpert® MTB/RIF testing. From sample receipt to test result release, the assessment covered everything, including internal quality control, lot to lot verification, method verification, equipment maintenance, competency assessment, and EQA activities. We used a standardized checklist depending on the testing services in the Diagnostic Centers. The questionnaire was created with the scope of service provided by TB diagnostic laboratories in mind (for TB culture laboratories and Xpert® MTB/RIF testing health facility laboratories).The previous records were reviewed and participant health facilities were interviewed. Prior to the data collection, training was given to the data collectors on the data collection tools.

Data management and analysis

A code number was assigned to each health facility participating in the study to ensure confidentiality. Data was entered and analyzed using SPSS version 25 (SPSS Inc., Chicago, Illinois, USA). The frequency and percentage of each variable were calculated. The table and figures were used for the presentation of the data.

Ethical clearance

The study was first approved by the Institutional review board (IRB) of the Ethiopian public health institute. Informed consent was collected from all participating health facilities before the interview. All study subjects have been informed of the purpose of the study and their right to anonymity. All the information obtained from the study subjects was coded to maintain confidentiality.

Results

General information on the testing laboratories service capacity

A total of 40 health facilities participated in this study, including nine governmental TB culture laboratories and thirty-one GeneXpert® MTB/RIF assay testing laboratories. Of all TB culture laboratories, three were also assessed for quality assurance practice of GeneXpert® MTB/RIF assay and AFB microscopy. Among the selected laboratories, 27/34 (79.4%) of GeneXpert® MTB/RIF assay testing laboratories and 7/9 (77.8%) of TB culture laboratories were enrolled in the SLMTA

program. Eventually, 1/27 (3.7 %) were accredited by the Ethiopian National Accreditation Office for AFB-microscopy and GeneXpert MTB/RIF assay, and 1/7 (14.2%) for TB culture and identification (ID) (Table 1).

Sample collection and transportation system

In this study, we assessed the pathway of quality assurance practice from sample reception to processing. More than half of Xpert® MTB/RIF testing laboratories, 19/34 (55.9%) and TB-culture laboratories, 6/9 (66.7%), had no sample collection date monitoring tools. A triple packaging system was used in 30/34 (88.2%) of all Xpert® MTB/RIF testing laboratories and all TB culture laboratories to receive samples from different facilities. In 8/9 (88.9%) of TB-culture laboratories, specimen rejection logbooks and rejection criteria were in place. However, in Xpert® MTB/RIF testing laboratories, only 21/34 (61.8%) of them had specimen rejection logbooks and rejection criteria as shown in Table 1. Moreover, 3/9 (33.3%) culture laboratories and 11/34 (32.4%) Xpert® MTB/RIF testing laboratories failed to monitor the temperature of the refrigerator used for sample storage. Also, three TB-culture laboratories and 24 Xpert® MTB/RIF testing laboratories did not calibrate their thermometers regularly. Moreover, almost 50% of TB-culture laboratories, 4/9 (44.4%) were no regular negative pressure calibration.

Availability of Standard operating procedure

This study also assessed the presence of the standard operating procedure (SOP) which is relevant in TB diagnostic laboratories. Of all laboratories, 24/34 (70.6%) of Xpert® MTB/RIF testing laboratories had internal quality control SOP for ZN staining techniques and 23/34 (67.6%) had internal quality control SOP for FM staining techniques. Among the TB-culture laboratories, 7/9 (77.8%) had IQC SOP for TB culture processing, 8/9 (88.9%) for in-house prepared media, and 6/9 (66.7%) for LPA. New reagent lot verification SOP in Xpert® MTB/RIF testing laboratories was available in less than 25% of the laboratories (17.6%) for both microscopy and Xpert® MTB/RIF assay and above 50% of TB-culture laboratories (55.6%), as illustrated in ~~(Table 3)~~

Quality assurance practice for AFB Smear microscopy testing

All Xpert® MTB/RIF testing laboratories performed AFB microscopy for diagnosis and TB treatment monitoring. Batch to batch QC testing of AFB reagents in 67.6% of the laboratories, IQC testing in 50%, and monitoring expiry dates of staining reagents in 70.4% of Xpert® MTB/RIF

testing laboratories were practiced (Fig 1). However, method verification for FM and /or ZN was not well-practiced (8.8%).

Quality assurance practice for GeneXpert® MTB/RIF assay

Among Xpert® MTB/RIF testing, only 29.4% monitored the Probe Check Control (PCC) and Specimen Processing (SPCC) (Fig 2). In 3/34 (8.8%) of the laboratories, a lot[illegible]lot verification of new cartridges was performed. Seven (20.6%) of the Xpert® MTB/RIF testing laboratories have performed the method verification. Annual GeneXpert machine calibration was performed in 20/34 (58.8%) of the Xpert® MTB/RIF testing laboratories. However, 11/34 (32.4%) of them run the samples with a machine that was not calibrated.

Quality assurance practice for TB-culture and identification

All TB-culture laboratories apply both MIGT and LJ media for TB-culture processing. During sample processing, a negative control was included with each batch and 3/9 (33.3%) of them included a positive control periodically. Internal quality control for the lateral flow assay (MPT64 Ag testing kit) was performed in 5/9 (55.6%) of the TB-culture laboratories. In this study, the number of TB-culture laboratories that verify lot-to-lot or batch-to-batch reagents ranged from 8/9 (88.9%) for MGIT media, and growth supplement and PANTA to 3/9 (33.3%) for the lateral flow assay. Method verification for the MGIT machine and MPT64 Ag testing was performed only in 3/9 (33.3%) of TB-culture laboratories (Table 3).

Quality assurance practice for Genotypic (LPA based) drug susceptibility testing

All nine TB-culture laboratory experts included positive and negative controls with each batch of specimens/cultures or one batch per week when LPA first and/or second-line performed. The laboratories also monitored the MTBDR*plus* assay and /or the MTBDRsl assay amplification control, conjugate control, TUB, locus control. New lot verification was performed in only 3/9 (33.3%) of TB-culture laboratories and 5/9 (55.6%) of them monitored the expired date and storage condition of kits (Table3).

External quality assurance practice in TB-diagnostic laboratories

Participating in EQA-blinded rechecking was observed in 18/34 (52.9%) of Xpert® MTB/RIF testing health facilities for AFB-microscopy where the majority of them (18/39%) participate quarterly. Also, PT-participation practice for AFB-microscopy and Xpert® MTB/RIF assay were

27/34 (79.4%) and 34 (100%) respectively. However, this study observed that there was no similar PT participation program. Among the participants, 19/34 (55.9) participated bi-annually in the Xpert® MTB/RIF assay. In TB-culture laboratories, 8/9 (88.9%) participated in PT and 9 (100%) in LPA. On-site supervision practice was not well practiced in TB-culture laboratories, only 3/6 (33.3) by comparison with the Xpert® MTB/RIF assay, which was 30/34 (88.2%) (Table 4).

Challenges of external quality assurance practice

This study identified 11/27 (40.7%), 8/27 (29.6%), and 9/27 (33.3%) of Xpert® MTB/RIF testing laboratories had more challenges with delayed PT-sample transportation, communication gap with the provider, and feedback missing respectively during AFB PT-participation. Major gaps identified during Xpert® MTB/RIF assay PT-participation were feedback missing 13/34 (38.2%) and delays in feedback from provider 12/34 (35.3%). The problem was a relative decline in TB-Culture Laboratories PT-participation, as indicated (Table 4).

Discussion

Implementation of quality assurance practice in TB laboratories plays a critical role in meeting the 2035 end-TB strategic goals (22). In our study, 7/9 (77.8%) TB-Culture laboratories and 27/34 (79.4%) of the Xpert® MTB/RIF testing laboratories were enrolled in the SLMTA program. According to the literature, the implementation of the SLMTA program resulted in the provision of quality-assured laboratory services for patient care (23) and assisted laboratories in becoming ISO 18519 accredited (24–31). During the study period, however, only 1/27 (3.7%) of Xpert® MTB/RIF testing laboratories were accredited by the Ethiopian National Accreditation Office using ISO 18519 standards and the AFB -Microscopic test among those enrolled in the SLMTA program. Similarly, only 1/7 (14.2%) of TB-Culture laboratories have achieved accreditation for TB-Culture and ID testing. Accreditation of laboratories based on the ISO15189 standard increases the functioning of laboratories in terms of monitoring and improving the entire testing process (32).

The Xpert® MTB/RIF machine has been used as the primary TB diagnostic test in Ethiopia and most Xpert® MTB/RIF testing laboratories serve as referral sites since the machines are not distributed to all health facilities (33). We observed that one-quarter of TB-culture laboratories and nearly half of Xpert® MTB/RIF testing health facilities didn't monitor the date of sample collation, which reduces the sample quality, which is an indicator of difficulty in tracing early problems before

processing. Studies have shown that the most frequent sources of errors that have occurred in laboratories are in the pre-pre- and pre-analytical phases that take up 48%-62% of total errors (34).

According to this study, 4/34 (11.8%) of Xpert® MTB/RIF testing health facilities received samples from health facilities that did not use standard triple packaging, which violated local and international sample transportation rules and regulations (35). It also disagrees with the WHO Laboratory Quality Management System handbook that, the laboratory should check sample quality, including its volume, container, and storage condition (5,16). The reference laboratory should keep track of the sample collection date for reception and must inspect the specimen transport box to ensure that specimens remain cool during transportation (18).

A poor sample does not ensure accurate results, while it is occasionally difficult to reject a sample (25,36). In this study, the practice of developing sample rejection criteria and recording the reason for rejection was more practiced in TB-culture laboratories comparing Xpert® MTB/RIF testing health facilities. The rejected sample shall be recorded in the rejection log book with the reason for rejection and shall be communicated with the requester (25,36). This will aid in the implementation of corrective and preventive measures if similar issues arise in the future.

The study participants' equipment calibration habits were poor in the majority of health facilities. For example, regular thermometry and GeneXpert machine calibration were performed only 10/34 (29.4%) and 20/34 (58.8%) of Xpert® MTB/RIF testing health facilities. A similar problem occurred in TB-culture laboratories. Only 5/9 (55.6%) of the TB-culture laboratories had experience of regular calibration of negative pressure, while negative pressure is the minimum design feature necessary to safely manipulate TB cultures (37). Performing regular preventive maintenance and calibration for each piece of equipment is important. That has a direct effect on the laboratory results (18).

This study observed that a lot-to-lot verification and method verification SOP were not well practiced in most Xpert® MTB/RIF assay and TB-Culture diagnostic laboratories. SOPs for each testing procedure in the laboratory are used to describe in detail the complete technique for performing tests to ensure consistent and reproducible results will be generated (38). Thus, all TB diagnostic health facilities are expected to have SOPs relating to their services.

Even though absolute accuracy is impossible to achieve in laboratory tests, experts need to consider all possible potential sources of error, including the quality of the stains and the staining procedure (39). In line with these, there are also international and national recommendations on requirements

for internal quality control, and reagent verification to be performed (18,39). In our findings, the number of health facilities that run the IQC of AFB smear is much higher (50%) than in a study conducted in Southern Ethiopia, 25.6% of tuberculosis laboratories (40) and Western Amhara (45%) of tuberculosis laboratories (8). The results of the outcome may vary because of study site differences. This study included only Xpert® MTB/RIF testing sites from all over the country.

This study also identified a small number of Xpert® MTB/RIF testing health facilities, which were observed monitoring internal controls, probe check control, and specimen processing control. The Xpert® MTB/RIF assay system automatically performs internal quality control (sample processing control and Probe Check Control) for each sample (40). While the machine is performing internal quality control, laboratory experts should monitor the machine's internal controls, probe check control, and Specimen Processing Control (41).

The use of a combination of both liquid and solid culture media gives the most optimal rates of mycobacterial recovery (42). Ethiopian TB-culture laboratories use both liquid and solid culture media. During TB-culture processing, 9 (100%) TB-culture laboratories included a negative (start and end) control and 3/9 (33.3%) of TB-Culture laboratories were running a positive control. The TB-culture laboratories should inoculate a negative (start and end) control in an MGIT tube and LJ tube to check for sterility of the processing reagents and monitor for cross-contamination and a positive sputum processing control (suspended pellet) to monitor NaOH killing of MTB (16).

According to this finding, TB testing method verification practices in Ethiopia's TB diagnostic laboratories were less than 50%, despite the fact that adhering to the new testing method is required to know the practice in a laboratory setup (16). Our finding showed that a lot-to-lot of verification practice for GeneXpert® MTB/RIF assay performed only in 8.8% of Xpert® MTB/RIF assay testing laboratories. Similarly, in TB-culture laboratories, lot-to-lot verification practices were much lower for LPA kits 3/9 (33.3%). Changing the reagent lots can adversely affect the consistency and quality of patient results. Therefore, it is recommended that the laboratory should take steps to evaluate and detect variation to reduce variation between lots at the point of manufacture (43).

Regarding EQA practice, this finding has shown that EQA-blind rechecking participation practice for AFB-Microscopy was 18/34 (52.9%) among Xpert® MTB/RIF testing health facilities. This finding is similar to the southern part of Ethiopia 9/13 (69.2%), which indicates there should be re-vitalization of the EQA-blind rechecking practice in the country (40).

In Ethiopia, TB diagnostic health facilities implement the three types of EQA program (blind rechecking, proficiency testing, and onsite supervision) (17,33). This assessment, however, identifies several challenges to EQA-PT participation; delays in the transportation of PT samples were a major issue in AFB-PT participation. In the study period, the PT samples were transported by the postal system or by TB focal person.

The communication gap with the provider is lower in TB culture laboratories compared to the GeneXpert® MTB/RIF assay. From the author's view, this may be due to the presence of few TB-Culture laboratories and having frequent contact with the provider for other work in addition to these activities. However, communication has not resolved the issue of missing EQA-feedback and delays in reporting to the provider, which requires further investigation and the establishment of a system for result delivery and reporting.

Limitation of the study

This study did not show the consistency or regular practice of quality assurance activities in health facilities. Most of the findings are not compared with other similar studies because of limited studies in the area. Rather, the findings are discussed with national and international guidelines recommended to have a quality-assured laboratory service.

Conclusions

Based on the assessment findings, IQC for AFB microscopy, TB culture and identification, and LPA were performed in most TB diagnostic health facility laboratories, while lot-to-lot verification and method validation were not practiced. Most Ethiopian TB diagnostic health facilities participate in the EQA-PT. However, there were difficulties during PT participation, including PT-transportation delays, communication gaps with the provider, delays in feedback from the provider, missing feedback, and reporting to the provider. Based on the finding, the authors strongly recommended performing internal quality control in all AFB smear microscopy testing health facilities, also performing a lot-to-lot verification for purchased reagents and batch to batch for in-house reagents. Additionally, method validation, regular machine calibration, and maintenance need critical consideration to be practiced. Furthermore, creating a system to avoid delays in feedback missing, result receiving, and delivery will solve the problem. Within this, the authors also recommend further studies be performed to identify the challenge of practicing quality assurance activities in TB diagnostic health facilities in depth.

Abbreviations

AFB: Acid-fast bacilli, DST: Drug susceptibility testing, EQA: External quality assessment, HC: Health Center, EPHI: Ethiopian Public, Health Institute, LPA: Line Probe Assay, LJ: Lowenstein Jensen, SLMTA: Strengthening Laboratory Management Toward Accreditation, MGIT: Mycobacterial growth indicator tubes, NTRL: National TB reference laboratory, QA: Quality assurance, QAS: Quality assurance System, QC: Quality Control, QMS: Quality management systems, SOP: Standard operating procedure, TB: Tuberculosis, PT: Proficiency testing, WHO: World Health Organization

Acknowledgment:

We would like to acknowledge the contribution of all the participant health facilities for their cooperation during the data collection process. We would like to extend our appreciation to all national TB research teams for their participation in the data collection.

Author Contributions

Conceptualization: Yeshiwork Abebaw.

Data curation: Yeshiwork Abebaw, Abebaw Kebede, Kirubel Eshetu, Ephrem Tesfaye, Mengestu Tadesse, Waganeh Sinshaw, Misiker Amare, Dinka Fikadu, Betselot Zerihun, Melak Getu, Getachew Seid, Anteneh Yalew, Getu Diriba.

Formal analysis: Yeshiwork Abebaw, Dinka Fikadu, Anteneh Yalew.

Funding acquisition: Yeshiwork Abebaw, Abebaw Kebede, Ephrem Tesfaye.

Investigation: Yeshiwork Abebaw, Abebaw Kebede, Kirubel Eshetu, Ephrem Tesfaye, Mengestu Tadesse, Waganeh Sinshaw, Misiker Amare, Dinka Fikadu, Betselot Zerihun, Melak Getu, Getachew Seid, Anteneh Yalew, Getu Diriba.

Validation: Yeshiwork Abebaw, Abebaw Kebede, Kirubel Eshetu, Anteneh Yalew.

Methodology: Yeshiwork Abebaw, Abebaw Kebede, Kirubel Eshetu, Mengestu Tadesse, Waganeh Sinshaw, Dinka Fikadu, Anteneh Yalew.

Software: Anteneh Yalew.

Writing-original draft: Yeshiwork Abebaw.

Writing – review & editing: Yeshiwork Abebaw, Abebaw Kebede, Kirubel Eshetu, Seid, Anteneh Yalew, Getu Diriba.

Funding: The authors received no specific funding for this work.

Competing interests: All authors have declared that no competing interests exist.

Data availability statement: All relevant data are within the manuscript.

Reference

1. Wand Ph. Laboratory Role in Tuberculosis Control. Wisconsin Medical Journal 2003;102(6).
2. World Health Organization. A Draft Global Strategy for TB Research and Innovation; report by the Director-General. WHO/GTB/ 2019;1–6.
3. Mesfin EA, Taye B, Belay G, Ashenafi A, Girma V. Factors Affecting Quality of Laboratory Services in Public and Private Health Facilities in Addis Ababa, Ethiopia. ~~Ejifcc.~~ 2017;28(3):205-23.
4. Sımsek H. ~~Mycobacterial Diseases~~ Requirement of Quality Assessment for Modern Tuberculosis Laboratory Services. *Mycobact Dis.* 2011;1(2):1–5.
5. Dave P V, Patel ND, Rade K, Solanki RN, Patel PG, Patel P, et al. Proficiency panel testing a reliable tool in external quality assessment of sputum smear microscopy services in Gujarat, India. I*ndian J Tuberc* . 2011;58(3):113–9.
6. World Health Organization. Laboratory Quality Management System: Handbook. World Health Organization 2011. ISBN 978 92 4 154827 4
7. Krleza JL, Celap I, Tanaskovic JV. Special issue : External Quality Assessment in Laboratory Medicine Review circumstances .~~Biochemia Medica~~ 2017;27(1):97–109.
8. Asemahagn MA. Journal of Medical Diagnostic Assessing the Quality of Tuberculosis Laboratory Services in Selected Public and Private Health Facilities in Western Amhara , Ethiopia. *J Med Diagn* Meth 2014, 3:2
9. Chapter 8. Control Q. Quality Assurance And Quality Control. IPCC Good Practice Guidance and Uncertainty Management in National Greenhouse Gas Inventories:1–17. https://www.ipcc-nggip.iges.or.jp/public/gp/english/8_QA-QC.pdf (Access date 10/8/2020)
10. World Health Organization. Framework of Indikators and Targets for Laboratory Strengthening under the End TB Strategy. World Health Organization. 2016;1:28.
11. Hillemann D, Hoffner S, Cirillo D, Drobniewski F, Richter E, Rüsch-Gerdes S. First Evaluation after Implementation of a Quality Control System for the Second Line Drug Susceptibility Testing of Mycobacterium tuberculosis Joint Efforts in Low and High Incidence Countries. *PLoS One*. 2013;8(10):1–7.
12. Nikolayevskyy V, Hillemann D, Richter E, Ahmed N, Van Der Werf MJ, Kodmon C, et al. External quality assessment for tuberculosis diagnosis and drug resistance in the European union: A five year multicentre implementation study. *PLoS One*. 2016;11(4):1–14.
13. Hiwotu TM, Ayana G, Mulugeta A, et al. Laboratory system strengthening and quality improvement in Ethiopia. *Afr J Lab Med*. 2014;3(2)

14. Forging Partnerships to Eliminate Tuberculosis: A Guide and Toolkit, are available from the Division of Tuberculosis Elimination's online ordering system at www.cdc.gov/tb.

15. Misganaw A, Abebe M, Lulie A. External Quality Assessment (EQA) of Randomly Blinded Rechecking Slides on TB AFB Microscopy Laboratories: A Retrospective Study, Addis Ababa, Ethiopia. *Am J* . 2016;1(2):9–15.

16. Palomino JC, Martin A, Portaels F, Cathode RM, Rüsch-Gerdes S, Pfyffer GE, et al. Evaluation of Automated BACTEC MGIT 960 System for Testing Susceptibility of. Mycobacteriology Laboratory Manual .*J Clin Microbio*l. 2007;37(3):607–10.

17. Kebede A. Guidelines for quality assurance of smear microscopy for guidelines for quality assurance of smear microscopy for tuberculosis diagnosis. Federal Ministry of Health. 2016.

18. GLI Practical Guide to TB Laboratory Strengthening. www.stoptb.org/wg/gli. (Access date 10/8/2020)

19. Gelaye AB, Hirut BM, Tesfaye DJ, Tirore LD. Assessment of Tuberculosis Laboratory Quality : A Multi Centre Descriptive Cross Sectional Study in Sidama Zone , Southern Ethiopia. 2017;6(2):67-72.

20. Agency CS. Projected-Population-of-Ethiopia-20112019.Central Statistical Agency. Available from https://www.statsethiopia.gov.et/population-projection/ (Access date 10/8/2020)

21 World Health Organization, World Health Organization (WHO). Service Availability and Readiness Assessment (SARA) An annual monitoring system for service delivery: Implementation Guide, version 2.2. Serv Availab Readiness Assess (SARA); An Annu Monit Syst Serv Deliv Implement Guid. 2015;104–42.

22. Kumar K, Kon OM. Diagnosis and treatment of tuberculosis : latest developments and future priorities. *Ann Res Hosp* 2017;1:37

23. JN. A comprehensive review of the SLMTA literature part 2: Measuring success. Afr J Lab Med. 2014;3(2):8

24. A Andiric LR, Massambu CG. One laboratory's progress toward accreditation in Tanzania. Afr J Lab Med. 2014;3(2)

25. G J, Attaining ISO 15189 accreditation through SLMTA: A journey by Kenya's National HIV Reference Laboratory. Afr J Lab Med. 2014;3(2).

26. Yao K, Luman ET, SLMTA Collaborating Authors. Evidence from 617 laboratories in 47 countries for SLMTA-driven improvement in quality management systems. Afr J Lab Med. 2014;3(2),

27. Masamha J, Skaggs B, Pinto I. Working toward a sustainable laboratory quality improvement programme through country ownership: Mozambique's SLMTA story. Afr J Lab Med. 2014;3(2).

28. Maruta T, Yao K, Ndlovu N, Moyo S. Training-of-trainers: A strategy to build country capacity for

SLMTA expansion and sustainability. Afr J Lab Med. 2014;3(2)

29. Shumba E, Nzombe P, Mbinda A, et al. Weighing the costs: Implementing the SLMTA programme in Zimbabwe using internal versus external facilitators. Afr J Lab Med. 2014;3(2).

30. Mothabeng D, Maruta T, Lebina M, Lewis K, Wanyoike J, Mengstu Y. Strengthening Laboratory Management Towards Accreditation: The Lesotho experience. Afr J Lab Med. 2011;1(1):1–7.

31. Ntshambiwa K, Ntabe-Jagwer W, Kefilwe C, Samuel F, Moyo S. Translating a National Laboratory Strategic Plan into action through SLMTA in a district hospital laboratory in Botswana. Afr J Lab Med. 2014;3(2)

32. Silva P. Guidelines for Establishment of Accreditation of Health Laboratories.WHO. 2007;49.

33. Federal Democratic Republic of Ethiopia Ministry of Health/Ethiopian Public Heath Institute. Ethiopian Public Heath Institute Implementation Guideline for GeneXpert MTB / RIF Assay in Ethiopia. 2014.

34. Plebani M. Diagnostic errors and laboratory medicine causes and strategies. *EJIFCC*. 2015; 26(1): 7-14.

35. World Health Organizetion. Guidance on regulations for the transport of infectious substances 2019 - 2020:1–56. Available from:https://apps.who.int/iris/bitstream/handle/10665/325884/WHO-WHE-CPI-2019.20-eng.pdf?uaWHO-WHE-CPI (Access date 10/8/2020)

36. Sample processing. Laboratory Quality Management System: World Health Organizetion . 64–5. Available from:https://extranet.who.int/lqsi/sites/default/files/attachedfiles/LQMS%205-4%20Sample%20Processing.pdf (Access date 10/8/2020)

37. World Health Organization .Tuberculosis laboratory biosafety manual. WHO/HTM/TB/2012.11

38. Akyar I. Standard Operating Procedures (What Are They Good For ?). Latest Research into Quality Control, Isin Akyar. IntechOpen,2012; Available from: https://www.intechopen.com/books/latest-research-into-quality-control/standard-operating-procedures-what-are-they-good-for(Access date 10/8/2020)

39. Association of Public Health Laboratories. External Quality Assessment for smear microscoy Programs NTB, Laboratories NR. A CE. https://www.aphl.org/aboutAPHL/publications/Documents/External_Quality_Assessment_for_AFB_Smear_Microscopy.pdf (Access date 10/8/2020)

40 . Shargie EB, Yassin MA, LindtjØrn B. Quality control of sputum microscopic examinations for acid fast bacilli in southern Ethiopia. Ethiop J Heal Dev. 2005;19(2).

41 Cephied. GeneXpert Dx System Operator Manual. 301-0045, Rev.2012

42. Practical Guide to Implementing a MTB/RIF Testing System for Xpert (“Xpert QA

Guide”). http://www.stoptb.org/wg/gli/assets/documents/Xpert-QA-guide-2019.pdf (acesed date17/July2020)

43. Range TN, Applications C. Mycobacterial Culture.*APHL*. 2011;20–1.

44. Thompson S, Chesher D. Lot-to-Lot. Mini-Review. *Clin Biochem*. 2018;39(2):51–60.

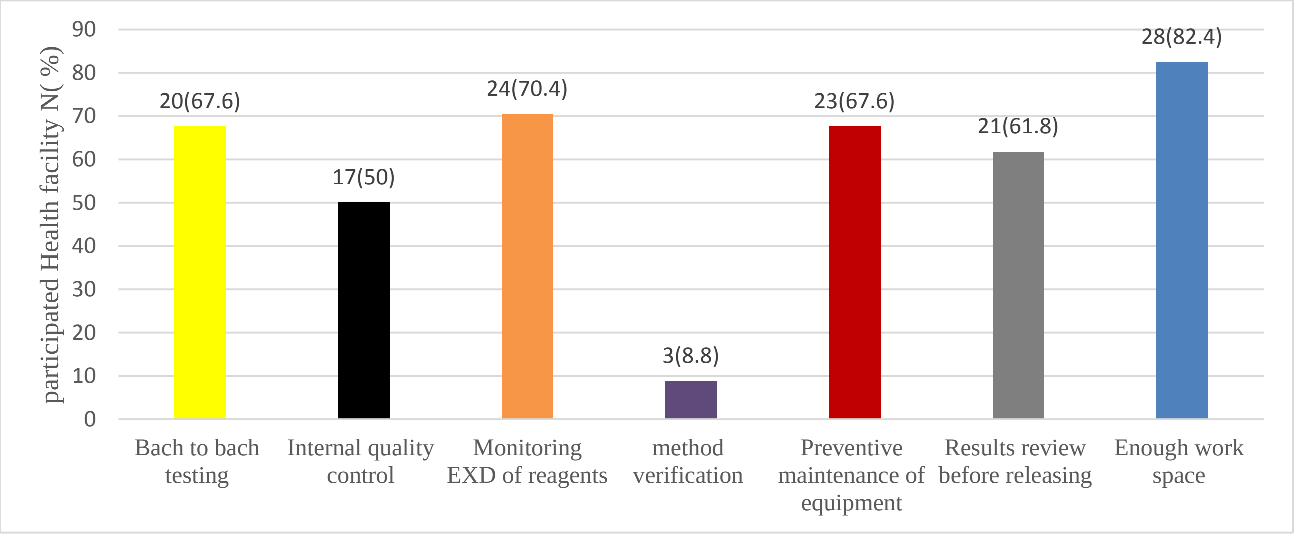

*EXD: Expired date

Figure 1:- Practice of quality assurance activities for microscopy

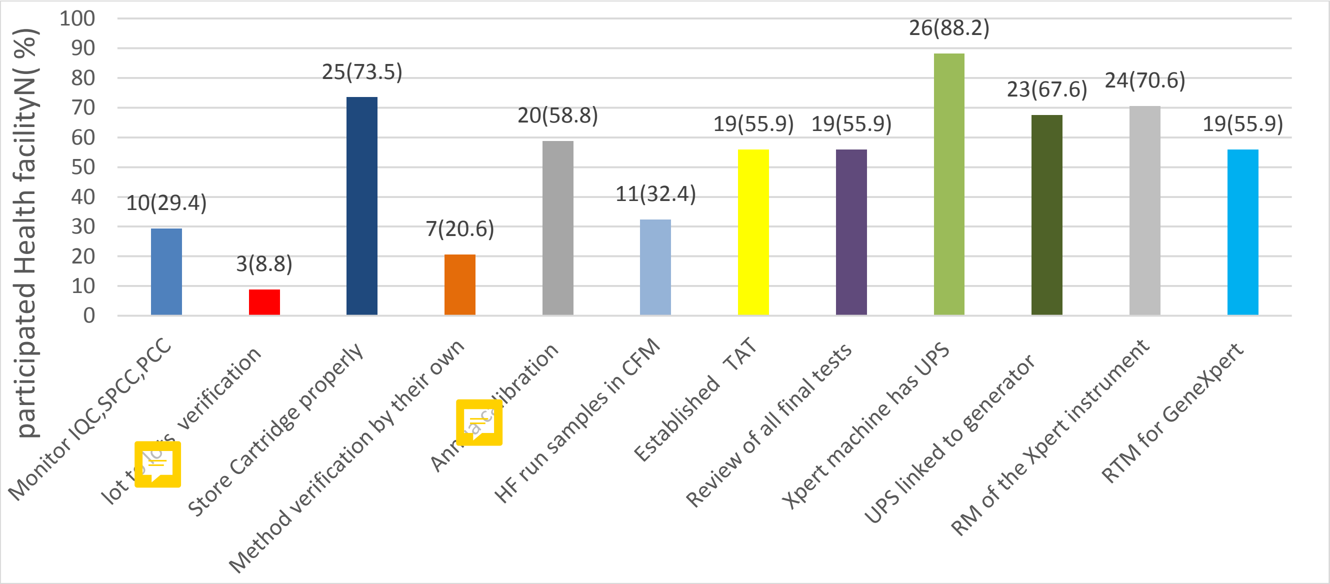

*HF-Health Facility, *CFM calibration failed Module *UPS- Uninterruptible power supply, *TAT-Turnaround time, * RTM-Room temperature monitoring, RM-Routine maintenance

Figure 1:- Pra[illegible]ce of quality assurance activities for Gene Xpert® MTB/RIF assay

Table 1: General information on quality assurance practice in TB culture laboratories and Xpert testing health facilities

			Gene Xpert® MTB/RIF assay testing health facilities n (%)	TB culture laboratories n(%)
Study sites		Health Center	8/34(23.5%)	0
		Hospital	24/34(70.5%)	2/9(22%)
		Institutional laboratory	2/34(6%)	7/9(78%)
Number of health facilities enrolled in SLIMTA program		Yes	27/34 (79.4)	7/9 (77.8)
		No	7/34 (20.6)	2/9 (22.2)
Accredited test				
AFB Microscopy		Yes	1/27(3.7)	NA
		No	26/27(96.3)	NA
GeneXpert MTB/RIF assay		Yes	1/27(3.7)	NA
		No	26/27(96.3)	NA
Culture and ID		Yes	NA	1/7(14.2)
		No	NA	6/7(85.7)
LPA	LPA1st line	Yes	NA	0
		No	NA	7(100)
	LPA 2nd line	Yes	NA	0
		No	NA	7(100)
Laboratory activities in 34 GeneXpert® MTB/RIF assay testing laboratory and nine TB-culture laboratories				
Monitor of the sample collection date to reception		Yes	19(55.9)	6(66.7)
		No	15(44.1)	3(33.3)
Ensuring sample transportation box kept cool during transport		Yes	19(55.9)	6(66.7)
		No	15(44.1)	3(33.3)
Way of specimens transportation	Mailing or courier system	Yes	25(73.5)	7(77.8)
		No	9(26.5)	2(22.2)
	Hand carry by staff	Yes	10(29.4)	5(55.6)
		No	24(70.6)	4(44.4)
	Hand carry by patient	Yes	4(11.8)	5(55.6)
		No	30(88.2)	4(44.4)
Use of standard triple packaging		Yes	30(88.2)	9(100)
		No	4(11.8)	0
Rejection log book and rejection criteria		Yes	21(61.8)	8(89.9)
		No	13(38.2)	1(11.1)
Separate refrigerator for sample storage		Yes	19(55.9)	8(89.9)
		No	15(44.1)	1(11.1)
Routine preventive maintenance of refrigerators		Yes	23(67.6)	6(66.7)
		No	11(32.4)	3(33.3)
Refrigerator temperature monitoring		Yes	23(67.6)	8(89.9)
		No	11(32.4)	1(11.1)
Thermometer calibration		Yes	10(29.4)	6(66.7)
		No	24(70.6)	3(33.3)

*LPA-Line probe Assay, *ID-Identification, *TB-Tuberculosis, *AFB-Acid fast bacillus, *QMS- Quality management system, *RIF- Rifampicin, *N- Number of participant, *NA- None applicable, *MTB-Mycobacterium Tuberculosis, *SLIMTA- Strengthening Laboratory Management toward Accreditation *TB-Tuberculosis, *n-Number of participant

Table 2: SOP avail for TB diagnostic tests among 34 Xpert testing health facilities and nine TB-culture laboratories in Ethiopia.

Selected SOP and log books		34 GeneXpert® MTB/RIF assay testing health facilities			Nine TB-culture laboratories		
		Microscopy n (%)		GeneXpert MTB/RIF assay n (%)	Culture and ID n (%)		LPA n (%)
		ZN	FM		Processed Samples	Prepard Media	
SOP for Internal Quality Control	Yes	24(70.6)	23(67.6)	31(91.2)	7(77.8)	8(88.9)	6(66.7)
	No	7(20.6)	6(17.6)	3(8.8)	2(22.2)	1(11.1)	3(33.3)
	NA	3(8.8)	5(14.7)	N/A	N/A	N/A	N/A
Method Verification	Yes	13(38.2)		18(52.9)	7(77.8)		6(66.7)
	No	21(61.8)		16(47.1)	2(22.2)		3(33.3)
Lot Verification for new reagents	Yes	6(17.6)		6 (17.6)	5(55.6)		5(55.6)
	No	28(82.4)		28(82.4)	4(44.4)		4(44.4)
Continuous Quality Improvement	Yes	22(64.7)		22(64.7	7(77.8)		7(77.8)
	No	12(35.3)		12(35.3)	2(22.2)		2(22.2)
Equipment Service Maintenance	Yes	20(58.8)		22(64.7)	7(77.8)		7(77.8)
	No	14(41.2)		12(35.3)	2(22.2)		2(22.2)
EQA Guideline/SOP	Yes	21(61.8)		18(52.9)	6(66.7)		6(66.7)
	No	13(38.2)		16(47.1)	3(33.3)		3(33.3)
Standard Request And Reporting Form	Yes	30(88.2)		30(88.2)	9(100)		9(100)
	No	4(11.8)		4(11.8)	0		0
Standard Registration Log Book	Yes	31(91.2)		27(79.4)	9(100)		9(100)
	No	3(8.8)		7(20.6)	0		0

* LPA-Line probe Assay, *ID-Identification, *TB-Tuberculosis, *RIF- Rifampicin, *N- Number of the participant, *MTB-Mycobacterium Tuberculosis, *ZN-Ziehl-Neelsen, *FM-fluorescence microscopy

Table 3: TB-culture laboratories those practiced quality assurances activates for TB-culture and identification among nine TB-culture laboratories of Ethiopia

Activities		Frequency and percent	
		Yes, n (%)	No, n(%)
Regular BSC calibration		9(100)	0
Regular negative pressure calibration		5(55.6)	4(44.4)
Include end and start control (negative control)		9(100)	0
Include a positive control		3(33.3)	6(66.7)
Perform lot to lot verification for media(MGIT tube), Growth Supplement, and PANTA		8(88.9)	1(11.1)
Monitor the storage conditions and expiration date of the lyophilized PANTA		5(55.6)	4(44.4)
Monitor expired date of reconstituted PANTA		7(77.8)	2(22.2)
Proper storage and monitoring of the expired date of MGIT reagent		4(44.4)	5(55.6)
Proper storage and monitoring of the expired date in-house reagent and media		7(77.8)	2(22.2)
Testing each new lot/batch of in-house prepared media for sterility, growth performance	NALC-NaOH,	5(55.6)	4(44.4)
	4%NaOH	5(55.6)	4(44.4)
	BHI/blood agar	4(44.4)	5(55.6)
	PBS buffer*(including PH)*	5(55.6)	4(44.4)
	LJ media	5(55.6)	4(44.4)
MGIT instrument preventive maintenance.		7(77.8)	2(22.2)
Routine preventive maintenance for incubator		6(66.7)	3(33.3)
Thermometry calibration for incubators		6(66.7)	3(33.3)
Annual MGIT machine calibration		7(77.8)	2(22.2)
MGIT machine method verified by your own laboratory		3(33.3)	6(66.7)
Recording growth results in weekly form LJ		9(100)	0
Preparing a blood agar plate for all positive cultures		8(88.9)	1(11.1)
Inspecting all negative cultures for growth prior to discarding.		8(88.9)	1(11.1)
Incubate negative result for a negative result for 8 weeks		9(100)	0
Confirm the presence of MTB vs NTM using Lateral flow assay		9(100)	0
Including positive and negative controls periodically for Lateral flow assay		5(55.6)	4(44.4)
Proper storage and monitoring of the expired kit		7(77.8)	2(22.2)
Lateral flow assay method verified by your laboratory		3(33.3)	6(66.7)
Re-incubating and retesting all BHI negative and Smear negative		7(77.8)	2(22.2)
Testing new lot numbers of Lateral flow assay kit		3(33.3)	6(66.7)
Review of all final test results by a second person		7(77.8)	2(22.2)
Avail UPS for Incubator		3(33.3)	6(66.7)
UPS avail for MGT machine		9(100)	0
QC for LPA			

IQC runs with each batch of specimens/cultures or one batch per week.		9(100)	0
Areas for master-mix preparation, DNA template addition/PCR, and detection are physically separated.		9(100)	0
For the MTBDRplus assay and /or MTBDRsl assay, MTB must be detected for INH and RIF results to be valid.	Amplification control	9(100)	0
	Conjugate control	9(100)	0
	TUB	9(100)	0
	Locus control	9(100)	0
For the MTBDR*plus* and /or MTBDR*sl* assays, if invalid results are obtained, repeating with newly extracted DNA from the same specimen. I		8(88.9)	1(11.1)
Develop LPA TAT	From sediment	9(100)	0
	From culture	9(100)	0
QC testing new lots of LPA kits with appropriate controls before using.		3(33.3)	6(66.7)
Monitoring of expired date and storage condition of kits		5(55.6)	4(44.4)
Routine maintenance and cleaning	Heat block,	5(55.6)	4(44.4)
	Thermal Cycler	5(55.6)	4(44.4)
	PCR hood	5(55.6)	4(44.4)
	TwinCubator	6(66.7)	3(33.3)
	Water bath	5(55.6)	4(44.4)
	Microcentrifuge	5(55.6)	4(44.4)
Calibration of Automatic pipette		6(66.7)	3(33.3)
Review of all final test results by a second person		6(66.7)	3(33.3)
UPS available for twincubetor		6(66.7)	3(33.3)

*MGIT- Mycobacteria growth indicator tube, *BHI- brain-heart Infusion, *LJ- Löwenstein–Jensen, *PBS- Phosphate-buffered saline, *NALC/NaOH- N-acetyl-l cysteine–sodium hydroxide, *NTM- Nontuberculous mycobacteria, *MTB-Mycobacterium Tuberculosis, *IQC- Internal quality control, *UPS- Uninterruptible power supply, *n=Number of participant, *PCR- Polymerase chain reaction, *DNA- Deoxyribonucleic acid, *MTBDR- Mycobacterium tuberculosis drug resistance, *INH- Isoniazid, *TAT- Turnaround time, *QC- quality control, *MTB-Mycobacterium Tuberculosis, *LPA-Line probe Assay, *RIF- Rifampicin, *UPS- uninterruptible power supply, *IQC- Internal quality control,

Table 4: External quality assurance practice in selected TB-diagnostic health facilities and TB-culture laboratories

Participation schemes		Microscopy n (%)	GeneXpertMTB /RIF assay n (%)	Culture and ID n(%)	LPA n (%)
Number of blind rechecking participant HF		18/34(52.9)	N/A	N/A	NA
Blind rechecking participation per year	Annually	3/18(16.75)	N/A	N/A	N/A
	Bi-annual	2/18(11.1)	N/A	N/A	N/A
	Quarterly	13/18(72.2)	N/A	N/A	N/A
PT participant HF		27/34(79.4)	34(100)	8/9(88.9)	9(100)
	Annually	3/27(12)	4/34(11.8)	4/8(50)	2/9(22.2)

PT participation per a year	Bi-annual	7/27(28)	19/34(55.9)	4/8(50)	4/9(50)
	Every fourth month	9/27(36)	4/34(11.8)	0	1/9(11.1)
	Quarterly	6/27(24)	6/34(17.6)	0	0
	Unknown	2/27(7.4)	1/34(2.9)	0	2/9(22.2)
Onsite supervision practice		30/34(88.2%)		3/6 (33.3)	
**EQA participation challenge among EQA participant of TB diagnostic health facilities**					
Leakage		NA	2/34(5.9)	0	0
PT sample volume		NA	2/34(5.9)	1(12.5)	0
Unclear procedure instruction		0	0	1(12.5)	1(12.5)
Commitment of staff		2(7.4)	4(11.8)	0	0
Interruption of PT sample		1(3.7)	7(20.6)	0	0
Delay in transportation		11(40.7)	4(11.8)	2(25.0)	3(33.3)
Communication gap with the provider		8(29.6)	10(29.4)	0	1(12.5)
Lack of standard request and reporting paper		2(7.4)	4(11.8)	O	0
Delays in feedback receiving		5(18.5)	12(35.3)	2(25.0)	3(33.3)
Feedback missing		9(33.3)	13(38.2)	0	
Delay in reporting		6(22.2)	9(26.5)	1(12.5)	3(33.3)

*RIF- Rifampicin, *NA- None applicable, *MTB-Mycobacterium Tuberculosis, * LPA-Line probe Assay, *ID-Identification, *PT- panel testing, n=Number of the participant, HF- Health Facility
