# Supplementary material for: Quality assurance practices in tuberculosis diagnostic health facilities in Ethiopia
Source: PLoS One. 2022 Jun 9;17(6):e0269601. doi: 10.1371/journal.pone.0269601 (PMC9182608; doi:10.1371/journal.pone.0269601)
Supplement: Attachment — Submitted filename: PLOS ONE REVIEW OCTOBER 2021.docx [file pone.0269601.s002.docx]

**PLOS ONE REVIEW OCTOBER 2021**

**QUALITY ASSURANCE PRACTICES IN TUBERCULOSIS DIAGNOSTIC HEALTH FACILITIES IN ETHIOPIA**

1. Line 86. The authors need to be clear of the difference between SLIPTA and SLMTA and ensure they are using these terms appropriately. See https://slmta.org/uploads/category_file/48/SLMTA%20VS%20SLIPTA.pdf
2. Line 96. LPA. All acronyms should be written out in full when first used, even if they are listed in the list of acronyms.
3. Line 102. The statement needs clarification: “…facilities lack the means to implement QC and participate in EQA…”. Can the reasons be stated here or in the discussion as to what means are needed, or otherwise what are the reason for facilities failing to implement QC and participate in EQA? See also point 14 below with respect to the conclusions.
4. Line 112. The statement needs clarity: “…second most populated country…”
5. Line 129, section “Data collection methods”. It is not clear if the checklist only collected retrospective data from records and from interviews with participants. Were any procedures actually observed during the assessment? If so, how was the Hawthorne effect avoided?
6. Line 141. The word “Data” is plural so should read “Data were…”
7. Lines 154 – 155. Can the authors clarify whether the 34 laboratories performing the GeneXpert MTB/RIF testing were also performing AFB microscopy? This is implied in Table 1, and should be stated in the narrative at this point.
8. Line 156. The word “Eventually” is misplaced here. It is assumed that these laboratories were accredited to a national or international standard at the time of the study. Can the authors explain more about the Ethiopian National Accreditation Office, which is also mentioned in Line 233; how it operates, and what is its ILAC status? Does the ENAO have the authority to accredit laboratories to international standards or does it use national standards?
9. Line 159, section on “Sample collection and transportation system”. Figures stated here that also appear in Table 1 need not be repeated in the narrative. Only main points from the table that the authors wish to bring out should be stated in the narrative; otherwise readers should be referred to Table 1. Similarly for Line 172, section on “Availability of Standard Operating Procedures”; Line 181, section on “Quality assurance practice for AFB smear microscopy testing”; and for the other results under the subsequent subheadings, in relation to the results presented in Tables 2 and 3.
10. Line 230–231. The sentence should read, for clarity: “According to the literature, the implementation of the SLMTA program *should result* in the provision of quality-assured laboratory services for patient care [23] and *assists* laboratories *to become* ISO…”. This tense is all important.
11. Line 254. Should read for clarity: “…was more practised in TB-culture laboratories *compared to* GeneXpert…”.
12. Lines 255–257: Should read, for clarity: “The rejected sample *should be* recorded… and should be communicated…. This *would* aid in the implementation...”.
13. Although the results describe how many laboratories practised various IQC methods, or participated in EQA schemes, the actual results of these practices or scheme participation are not given. How many laboratories performed well in IQC or in EQA? Did any laboratories fail in IQC or EQA? These data should be available from the records and would provide objective evidence of performance.
14. Line 319. Conclusions. What other measures could be put in place to promote better practices? What are the challenges with the supply systems and equipment maintenance services and what is needed to improve them? There is also little discussion on on-site supervisory practices and how they could be enhanced. Further studies are suggested, but from this study there are already points of evidence to propose preliminary measures to improve and sustain the quality of diagnostic practices for tuberculosis.
15. Table 1. Please also refer to point 8 above. “Accredited test” needs explanation.

Jane Carter

Amref Health Africa
