# Supplementary material for: Quality assurance practices in tuberculosis diagnostic health facilities in Ethiopia
Source: PLoS One. 2022 Jun 9;17(6):e0269601. doi: 10.1371/journal.pone.0269601 (PMC9182608; doi:10.1371/journal.pone.0269601)
Supplement: Attachment — Submitted filename: Response to Reviewers.docx [file pone.0269601.s003.docx]

**Ref: PONE-D-21-2092**

**QUALITY ASSURANCE PRACTICES IN TUBERCULOSIS DIAGNOSTIC HEALTH FACILITIES IN ETHIOPIA**

Dear Editor,

Thank you for your informative comments and thoughtful comments on our manuscript. We have addressed your and the reviewers comments one by one. We also appreciate the reviewers for their critical observation and informative comments which radically improved our manuscript quality. We have provided point-by-point response to the reviewers' comments below with table. Also, we would like to inform you that we have used hyperlink to indicate where we made changes in the previous version of the manuscript based on the reviewers’ comments.

Responses to Editorial manager

| No | Editorial manager comment | Responses to Editorial manager |
| --- | --- | --- |
| 1 | Line 170-171- 'Moreover, almost 50% of TB-culture laboratories, 4/9 (44.4%) were no regular negative pressure calibration'. In this sentence, 'were' should be replaced by 'did not have' | Thank you very much for your critical observation. We have corrected accordingly on line 229 |
| 2 | Reviewer 1 has already pointed out that there is no Figure 2 which is mentioned in the text whereas Table 2 is missing from the main text. | Thank you very much for your insightful observation, we apologize for the error, it was a labeling problem for both Figure 2 and Table 2. Now it is corrected as Figure 2 which is mentioned in text line 563 and Table 2 in the text in line 204 |
| 3 | Line 232, 233- ISO Number to be corrected | Thank you very much for your insightful comments, we apologize for the error and have corrected it in line 262 |
| 4 | Line 241- 'collation' should be 'collection' | Thank you very much for your critical observation and we apologize for the error, we have corrected it in line 275 |
| 5 | Line 244- 'pre-pre and pre-analytical should be corrected. | Thank you very much for your observation we have corrected it in line 276 |
| 6. | Table 1- SLIMTA to be replaced by SLMTA | Thank you so much, we apologize for the error and have corrected it in Table 1 |

**Responses to reviewer 1**

| No | Reviewer 1 comment | Responses to reviewer |
| --- | --- | --- |
| 1 | First of all, you should check some sentences in terms of writing language. For example; In 154. line; Among the selected laboratories, 27/34 (79.4%) of GeneXpert®.....You can say 27 (79,4%)Gene Xpert Lab..... | Thank you for your insightful comments and we apologize for the error. We have corrected the errors in lines 172,173 and other similar mistakes |
| 2 | There are 2 kinds of Figure 1, but no figure 2 (see 490. and 495. lines!). | Thank you very much for your critical observation and we apologize for the error, it was a labeling problem. Now it is corrected Figure 2 in line 563 |
| 3 | You should write "Figure" do not abbreviate it, like Fig! | Thank you for your suggestion, We have corrected it in lines 209 and 214 |
| 4 | It is not required Method verification for FM or ZN. But you should check QC for staining solutions, slide preparation, and evaluation. So for all, there are negative- and positive- slide controls known results! The technician should be run positive and negative slide control in every work, and record their results. The result is okay, then should be continued patient slides. | Thank you very much for your critical observation. We have seen that 3 health facilities were verified FM or ZN method with 20 negative and 20 smear-positive slide but as you said, it is not required(has waived for method validation) because of several factors for verification so we have accepted the comment and removed it from the paper in line 210 and figure 1 |
|  | Your sample size for TB labs is written 34 +9 (43). But in 151. line, it is written totally of 40 labs. | We apologize for the confusion, as indicated in the result part line 168, a total of 40 health facilities were included in the study where six health facilities for TB-culture and LPA only, three health facilities for TB-culture, LPA, GeneXpert® MTB/RIF assay, and Microscopy testing, and thirty-one health facilities for GeneXpert® MTB/RIF assay and Microscopy only. This is also now mentioned in the sample size calculation part line 132 |
| 6. | Table 2 should be placed in text prior to Table 3. There is no Table 2 in text, except separate a Table 2! | Thank you for your insightful observation. We apologize for the error, it was a labeling problem with Table 3. Now it is corrected as Table 2 in the text in line 204. |
| 7 | In 189. line, ...a lot of lot verification....please correct this! | Thank you very much for your observation, We have corrected it in line 214 |
| 8 | In 217 line, On-site supervision practice was not well practiced in TB-culture laboratories, only 3/6 (33.3)....As sample size, it is written 9 TB- culture labs! But here is 6 labs! | Thank you very much for your critical observation. We have corrected it in line 247 |
| 9 | In 288. line, negative (star and end ) control....please corrected star as start. | Thank you very much for your critical observation. We have corrected it in line 324 |
| 10 | In 509. line, correct avail as available. | Thank you very much for your observation, We have corrected it in line 557 |
| 11 | In 490. line, Figure 1; lot to lots and annua.....please correct the. | Thank you very much for critical observation, We have corrected it finger 2 |
| 12 | In 365. line; references; Please check "reference writing rules" from guidelines for authors; ; Is journal writing stile italic or not! | Thank you very much for your comment, We have accepted and corrected it in line 461 |

**Responses to reviewer 2**

| No | Reviewer Comment | Responses to reviewer |
| --- | --- | --- |
| 1. | Line 86. The authors need to be clear of the difference between SLIPTA and SLMTA and ensure they are using these terms appropriately. | Thank you for your insightful comments and suggestions. Based on your comments, we have to rewrite it again in line 90. |
| 2 | Line 96. LPA. All acronyms should be written out in full when first used, even if they are listed in the list of acronyms | Thank you very much for your observation. We have corrected it in line 102. |
| 3 | Line 102. The statement needs clarification: “…facilities lack the means to implement QC and participate in EQA…”. Can the reasons be stated here or in the discussion as to what means are needed, or otherwise what are the reason for facilities failing to implement QC and participate in EQA? See also point 14 below with respect to the conclusions. | Thank you very much for your insightful comments and suggestions. Based on your comments, rephrase the sentence in 106-109 |
| 4 | Line 112. The statement needs clarity: “…second most populated country…” | Thank you very much for your observation, we have corrected it as “second-most populous nation” in line 119. |
| 5 | Line 129, section “Data collection methods”. It is not clear if the checklist only collected retrospective data from records and from interviews with participants. Were any procedures actually observed during the assessment? If so, how was the Hawthorne effect avoided? | Thank you for your critical observation. We observed the previous data to see the implementation (the present and absence laboratory recorded ) depending on the question now it is clearly stated in line 141 |
| 6. | Line 141. The word “Data” is plural so should read “Data were…” | Thank you very much for your observation. We apologize for the error and corrected accordingly on line 158 |
| 7 | Lines 154 – 155. Can the authors clarify whether the 34 laboratories performing the GeneXpert MTB/RIF testing were also performing AFB microscopy? This is implied in Table 1 and should be stated in the narrative at this point. | GeneXpert® MTB/RIF testing laboratories represent both GeneXpert® MTB/RIF assay and Microscopy testing laboratories so know We have accepted the comment and corrected on line 157 |
| 8 | Line 156. The word “Eventually” is misplaced here. It is assumed that these laboratories were accredited to a national or international standard at the time of the study. Can the authors explain more about the Ethiopian National Accreditation Office, which is also mentioned in Line 233; how it operates, and what is its ILAC status? Does the ENAO have the authority to accredit laboratories to international standards or does it use national standards? | Thank you very much for your comment. base done your comment we deleted “Eventually” and replaced by “however “ in line 173 and we state about statues ENAO in discussion part in line 267 |
| 9. | Line 159, section on “Sample collection and transportation system”. Figures stated here that also appear in Table 1 need not be repeated in the narrative. Only main points from the table that the authors wish to bring out should be stated in the narrative; otherwise readers should be referred to Table 1. Similarly for Line 172, section on “Availability of Standard Operating Procedures”; Line 181, section on “Quality assurance practice for AFB smear microscopy testing”; and for the other results under the subsequent subheadings, in relation to the results presented in Tables 2 and 3. | Thank you very much for your critical comments and suggestions. we have corrected table 1-3 and figure 1 with narrative text in lines 183-187 for Table 1 and line 192-197 and table 332-36.figur 1 line 205 |
| 10 | Line 230–231. The sentence should read, for clarity: “According to the literature, the implementation of the SLMTA program should result in the provision of quality-assured laboratory services for patient care [23] and assists laboratories to become ISO…”. This tense is all important. | Thank you very much for your insightful comments, we apologize for the error and have corrected it in line 261 |
| 11 | Line 254. Should read for clarity: “…was more practiced in TB-culture laboratories compared to GeneXpert…”. | Thank you very much for your suggestion. But based on your previous comment of number 9, it is removed from paper during the narrative |
| 12 | Lines 255–257: Should read, for clarity: “The rejected sample should be recorded… and should be communicated…. This would aid in the implementation...”. | Thank you very much for your suggestion. But based on your previous comment of number 9, it is removed from paper during the narrative |
| 13 | Although the results describe how many laboratories practised various IQC methods, or participated in EQA schemes, the actual results of these practices or scheme participation are not given. How many laboratories performed well in IQC or in EQA? Did any laboratories fail in IQC or EQA? These data should be available from the records and would provide objective evidence of performance. It is out of the scope of the objective | Thank you very much for your critical observation. Initially we need to see the trend of PT but because of incompleteness of data at the health facilities we excluded it and aimed to see only their practice. we appreciate your observation and put it as our limitation in line 363 |
| 14 | Line 319. Conclusions. What other measures could be put in place to promote better practices? What are the challenges with the supply systems and equipment maintenance services and what is needed to improve them? There is also little discussion on on-site supervisory practices and how they could be enhanced. Further studies are suggested, but from this study, there are already points of evidence to propose preliminary measures to improve and sustain the quality of diagnostic practices for tuberculosis. | Thank you very much for your observation again, however. This study missed addressing challenges for the implementation of internal quality control, lot to lot verification, method verification, equipment maintenance.  We suggested further identification of challenges since it is broad and need in-depth analysis. This study observes only the challenge of EQA with a specific area. Regarding on-site supervisory practices, we added more in line 344 |
| 16 | Table 1. Please also refer to point 8 above. “Accredited test” needs explanation. | Thank you very much for your critical observation and We apologize for the error, it was from health facilities enrolled in the SLMTA program, not from total health facilities, now we correct it in table 1 and result line 174 and discussion part line 264 |

Responses to reviewer 3

| No | Reviewer 3 comment | Responses to reviewer |
| --- | --- | --- |
| 1 | Authors mentioned the numbers of participants lab @ 43 at some time and 34, 40 at others. So they can clarify it | We apologize for the confusion, as indicated in the result part line 164, a total of 40 health facilities were included in the study where six health facilities for TB-culture and LPA only, three health facilities for TB-culture, LPA, GeneXpert® MTB/RIF assay and Microscopy testing, and thirty-one health facilities for GeneXpert® MTB/RIF assay and Microscopy only. This is also now mentioned in the sample size calculation part line 132 |
| 2 | Is it ISO 18519 or ISO 15189? Authors mentioned both | Thank you very much for your insightful comments, we apologize for the error and have corrected it in line 262 |
| 3 | Recommendation on line 297-298 are ambiguous. It is very unclear | Thank you very much for your critical comment. based on your comment have rephrased the sentence in lines 333-335 |
| 4 | Authors mentioned the checklist on line133. I would like to have a look at this | Thank you very much for your insightful observations. We have attached the checklist below this table |
| 5 | Paragraph@ lines 303-307 should in line with the standard format of "discussion". Here, it is uncertain about final messages | Thank you very much for your critical comment and We apologize for the confusion. We have removed the confusion sentence in lines 306-309 |
| 6. | Lines @ 320-323 sound contrary to lines @ 50-52 | Thank you for your critical observations and informative comments. We have corrected the conclusion part line 367. |

**Ethiopian Public Health Institute, National Tuberculosis Reference Laboratory**

**Assessment of Quality Assurance Practice in Tuberculosis Diagnostic Facilities of Ethiopia, 2018/19**

1.1. General information

| Q.NO | Questions | | Responses | | Skips Pattern | |
| --- | --- | --- | --- | --- | --- | --- |
| Section A. Identification | | | | | | |
| 1 | Full name of the interviewer | | | ______________________________ | |  |
| 2 | Phone number of the interviewer | | |  | |  |
| 3 | Name of interviewee | | | _________________________ | |  |
| 4 | Position and signature of interviewee | | | ______________________________ | |  |
| 5 | Date of interview (GC) | | | _____/_____/_________ DD/MM/YYYY | |  |
| 6 | Name of Health Facility: | | | ______________________________ | |  |
| 7 | Cluster code : Health Facility Code | | |  | |  |
| 8 | Address of health facility | Region | |  | |  |
|  |  | Zone/Sub City | |  | |  |
|  |  | Woreda | |  | |  |
|  |  | Kebele | |  | |  |
| 9 | Type of TB-test done in the laboratory | | | AFB Microscopy ………….……1  GeneXpert MTB/RIF Assay…2  Culture and Identification …3  LPA1st line ………….……4  LPA 2^nd^ line ………….…5  1^st^ line Phenotypic DST….6  2^nd^ line Phenotypic DST….7 | |  |
| 10 | Have your laboratory ever been involved in SLMTA program | | | Yes………….……1  No ………….……2 | |  |
| 11 | How many of your staff taken complete cohort of SLMTA training? (*No of training taken/total staff*) | | |  | |  |
| 12 | Do you have accredited test/s that used for TB diagnosis | | | AFB Microscopy ………….……1  GeneXpert MTB/RIF Assay………….……2  Culture and ID …….…3  LPA1st line ………….……4  LPA 2^nd^ line ………….……5  We have no accredited test …6 | |  |

## 1.2. Specimen Collection, Transport, and Laboratory Receipt Checklist

| A. **Instruction for laboratory quality assurance practice assessment.**   Indicate “Yes (Y),” “No (N),” or “not Applicable (NA)” for each response below. | | | | | |
| --- | --- | --- | --- | --- | --- |
| **SOP No.** | **SOP Title(*Observe and read to see the compliance of SOP with GLI guide line )*** | **Y** | **N** | **NA** | **Comment** |
|  | SOP For Sample Collection, Handling, Transport And Reception(*for both extrapolumonary or,and plumonary*) |  |  |  |  |

| B. **Instruction for laboratory quality assurance practice assessment.**   *Indicate “Yes (Y),” and “No (N)” for each response below.* | | | | |
| --- | --- | --- | --- | --- |
| **Key Elements** | | | **Y** | **N** |
| 1. Do you monitor the sample collection date to reception? (*observe registration, or sample collection or any other method of monitoring system with document evidence )* | | |  |  |
| 2. Check specimen transport box to ensure specimens were kept cool during transport *(observe monitoring log sheet)* | | |  |  |
| 3. Way of specimens transportation from other site *(Integrated Specimen Referral Network for Ethiopia* | Using dedicated mailing or courier system (Eg; Postal system-ground or air transport) | |  |  |
|  | Hand carry by staff and using public transport measures | |  |  |
|  | Hand carry by patient and using public transport measures | |  |  |
| 4. Are specimens packaged appropriately according to local and or international regulations and transported to referral laboratories (triple packaging) | | |  |  |
| 5. Do you have specimen rejection log book ?(*observe the log book)* | | |  |  |
| 6. Is sample volume Check(observe 3 month data from *specimen rejection log, or registration log book )* | | |  |  |
| 7. Is sample quality Check(observe 3 month data from specimen rejection log book, or registration log book ) | | |  |  |
| 8. Do you use standard sterile(clean for Microscopy) graduated falcon tube*?(observe falcon tube during study period )* | | |  |  |
| 9. Do you use separate refrigerator for sample storage and reagent storage?(*observe sample storage condition )* | | |  |  |
| 10. TAT of Sample reception to processing at referral laboratory (3 month data *registration log book*) | | |  |  |
| 11. Routine maintenance and cleaning of all equipment   (*Refrigerator, autoclave, deep freezer etc.)(observe 3 month data)* | | Check compressor |  |  |
|  |  | Record temperature |  |  |
|  |  | Clean exterior surfaces |  |  |
|  |  | Defrost and clean interior |  |  |
| 12. Calibration | | Refrigerator, |  |  |
|  |  | deep freezer |  |  |
|  |  | Temperature |  |  |
| 13. Are samples store until result reported (sample disposal log and storage area during study period) | | |  |  |

1.3. **Smear Microscopy Checklist**

| A. **Instruction for Relevant Standard Operating Procedures (SOPs)** **.**Indicate “Yes (Y),” “No (N),” or “not Applicable for (NA)” for each response below. | | | | | |
| --- | --- | --- | --- | --- | --- |
| **SOP No.** | **SOP Title(*Observe and read to see the compliance of SOP with GLI guide line )*** | **Y** | **N** | **NA** | **Comment** |
|  | SOP For Internal quality control for ZN |  |  |  |  |
|  | SOP For Internal quality control for FM |  |  |  |  |
|  | EQA guideline/SOP |  |  |  |  |
|  | SOP For Method Verification |  |  |  |  |
|  | SOP For New lot verification |  |  |  |  |
|  | SOP for Continuous Quality Improvement |  |  |  |  |
|  | SOP for equipment service maintenance |  |  |  |  |
|  | SOP for analytical and post analytical |  |  |  |  |
|  | Standard request and reporting form |  |  |  |  |
|  | Standard registration log book |  |  |  |  |

| B. **Instruction for laboratory quality assurance practice assessment.**   *Indicate “Yes (Y),” “No (N),” for each response below.* | | | | | | | | |
| --- | --- | --- | --- | --- | --- | --- | --- | --- |
| **Key Elements** | | | | | | | **Y** | **N** |
| 1. Including positive and negative smears with new batch of staining reagents or new preparation of staining reagents*.(* *by taking one result and back to the log book of batch to batch verification also observe at least 3 batch of reagent in lot to lot verification log book to know the continuity)* | | | | | | |  |  |
| 2. Including positive and negative smears with each batch of patient slides or weekly *(by taking one result and back to the log book also observe 3 month of quality control log book to know the continuity)*. | | | | | | |  |  |
| 3. Recording batch numbers and expiry dates of staining reagents used.(observe the label of current bottle that show batch numbers and expiry dates ) | | | | | | |  |  |
| 4. Is you laboratory established inventory management system (observe 3 month data) | | | | IFRR and RRF | | |  |  |
|  |  |  |  | Bincard | | |  |  |
|  |  |  |  | Is FIFO/FEFO applied | | |  |  |
| 5. Is FM and ZN method verified *(observe verification log that show the sensitivity and specificity of method )* | | | | | | |  |  |
| 6. Maintaining equipment (microscope)(observe 3 month data) | | | Daily(Remove dust with a brush , Replace Cover) | | | |  |  |
|  |  |  | Monthly *(cleaning of lens and stage of microscopy )* | | | |  |  |
| 7. Preparing smears and drying slides are performed in a Class II BSC if it is from sediment (observe work area) | | | | | | |  |  |
| 8. Preparing smears and drying slides are performed in ventilated area if it is direct sputum (observe work area). | | | | | | |  |  |
| 9. Labeling each slide with patient identification and specimen number (Observe the archived slide). | | | | | | |  |  |
| 10. Review of all final test results by a second person to minimize reporting errors*(observe duty roster, request form or any other aids )* | | | | | | |  |  |
| 11. Reporting results according to the WHO/IUALTD grading scale as per the Global Laboratory Initiative Sputum Microscopy Handbook*.(observe registration)* | | | | | | |  |  |
| 12. Reporting results to the clinic within 8 working hours of sputum receipt in the laboratory*.(observe registration and TAT monitoring log )* | | | | | | |  |  |
| 13. Is work area clean and have enough work space (*observe daily cleaning log and separate work area for registration staining and microscopy reading* ) | | | | | | |  |  |
| 14. Do you participate in Proficiency Testing(If yes skip to Q.16) | | | | | | |  |  |
| 15. If no why?*(If no write the reason and skip to Q.28)* | | | | | | |  |  |
| 16. Staff participation in PT panel testing (observe the duty roster, registration book on the day of TP result report) | | | | | | |  |  |
| 17. Testing EQA panels in the same manner as clinical specimens(observe the duty roster, registration book on the day of TP result report) | | | | | | |  |  |
| 18. If yes where did you get the PT samples? | | | | | IQAS | |  |  |
|  |  |  |  |  | NQAS | |  |  |
|  |  |  |  |  | RQAS | |  |  |
| 19. Did you have an agreement with PT provider? (observer the agreement or registration evidence) | | | | | | |  |  |
| 20. Is the PT provider accredited with ISO/IEC 17043?(observe the certificate if Unknown write UK) | | | | | | |  |  |
| 21. How many times do you receive the PT sample in a year( One per year, Bi annual, Quarterly)write on the comment section | | | | | | |  |  |
| 22. When did you start Smear PT participation (*write on the comment section*) | | | | | | |  |  |
| 23. What was your past microscopy PT performance (2017-2018 if available?) (*write on the comment section*) >85% EQA panels are passed | | | | | | |  |  |
| 24. Did you take investigation and action/s for all unacceptable performance? Do you implement corrective action for unacceptable PT performances? (*observe the document* ) | | | | | | |  |  |
| 25. What problem you face during PT slide reading | | | | | | |  |  |
| 26. Challenge of PT-EQA participation | Commitment of staff | | | | | |  |  |
|  | accessibility of PT samples | | | | | |  |  |
|  | interruption of PT sample | | | | | |  |  |
|  | cost of PT sample | | | | | |  |  |
|  | Donor funding interruption | | | | | |  |  |
|  | Others (specify) | | | | | |  |  |
| 27. Challenges during PT sample receiving and reporting? | Shipping method | | | | | |  |  |
|  | Delay in transportation*(Estimation of time Delay)* | | | | | |  |  |
|  | Communication gap with provider | | | | | |  |  |
|  | Lack of standard request and reporting paper | | | | | |  |  |
|  | Delays in feedback receiving*(Estimation of time Delay)* | | | | | |  |  |
|  | Feedback missing | | | | | |  |  |
|  | Delay in reporting *(Estimation of time Delay)* | | | | | |  |  |
|  | Other | | | | | |  |  |
| 28. Are you regularly supervised by regional or national TB supervisor | | | | | | National lab |  |  |
|  |  |  |  |  |  | Regional lab |  |  |
|  |  |  |  |  |  | NGO(challenge TB) |  |  |
| 29. If yes ,How many times the supervision done in a year(*1 per year, Biannual, Quarterly )* (*write on the comment section*) | | | | | | National lab |  |  |
|  |  |  |  |  |  | Regional lab |  |  |
|  |  |  |  |  |  | NGO(challenge TB) |  |  |
| 30. Is the supervisors use check list during supervision? | | | | | | |  |  |
| 31. Did you take corrective actions for the identified gaps during supervision? | | | | | | |  |  |
| 32. Do you participate blind rechecking for AFB microscopy? | | | | | | |  |  |
| 33. If yes How many times do you participate in blind rechecking in a year | | | | | | |  |  |
| 34. Are all slides are stored sequentially (observe the slide storage condition ) | | | | | | |  |  |
| 35. Are Slides selected according to the EQA guide line(observe copy of EQA slides sending format ) | | | | | | |  |  |
| 36. Is there any challenge in participating of blind rechecking for AFB microscopy? (1 per year, Biannual, Quarterly ) (write on the comment section) | | | | | | |  |  |
| 37. Have you an agreement with your referral sit to participate EQA with blind rechecking? | | | | | | |  |  |
| 38. What are the challenges during Blind rechecking smear sending and reporting? | | shipping method(slides may crack | | | | |  |  |
|  |  | slides are not properly stored | | | | |  |  |
|  |  | Lack of standard request and reporting paper | | | | |  |  |
|  |  | Negligence of staff to do PT samples | | | | |  |  |
|  |  | delay in reporting(Estimation of time Delay) | | | | |  |  |
|  |  | Delays in feedback receiving(*Estimation of time Delay)* | | | | |  |  |
|  |  | Result transcription error during sending to referral site | | | | |  |  |
|  |  | Other | | | | |  |  |
|  |  | Stock out | | | | |  |  |

1.4. **Cepheid Xpert® MTB/RIF Checklist**

| A. **Instruction for laboratory quality assurance practice assessment.** *Indicate “Yes (Y),”and “No (N),” or NA” for each response below.* | | | | | |
| --- | --- | --- | --- | --- | --- |
| **SOP No.** | **SOP Title(*Observe and read to see the compliance of SOP with GLI guide line )*** | **Y** | **N** | **P** | **Comment** |
|  | EQA guideline/SOP |  |  |  |  |
|  | SOP For Method Verification |  |  |  |  |
|  | SOP For New lot verification |  |  |  |  |
|  | SOP for Continuous Quality Improvement |  |  |  |  |
|  | SOP for equipment service maintenance |  |  |  |  |
|  | SOP for analytical and post analytical Including Internal quality control of Xpert® MTB/RIF |  |  |  |  |
|  | Standard request and reporting form |  |  |  |  |
|  | Standard registration log book |  |  |  |  |

| **B. Instruction for laboratory quality assurance practice assessment.** *Indicate “Yes (Y),” “No (N),” or for each response below. .* | | | | | | | | |
| --- | --- | --- | --- | --- | --- | --- | --- | --- |
| **Key Elements** | | | | | | | **Y** | **N** |
| 1. Is the Results of internal controls, Probe Check Control, and Specimen Processing Control monitored.(observe by taking one result and back to the monitoring log book and GeneXpert machine also observe 3 month of quality control monitoring log book to know the continuity) | | | | | | |  |  |
| 2. Is lot to lots of cartridges are tested with External Run Controls *(* *by taking one result and back to GeneXpert machine also observe at least 3 lot of reagent in lot to lot verification log book to know the continuity)* | | | | | | |  |  |
| 3. Routine maintenance of the GeneXpert instrument and refrigerator (*observe 3 month GeneXpert routine maintenance).* | | Daily *(Clean all module door )* | | | | |  |  |
|  |  | Monthly (*Archive results on a CD ,Purge tests Replace fan filters)* | | | | |  |  |
|  |  | Room temperature monitoring | | | | |  |  |
| 4. Not using expired and not properly stored Cartridge .(*check 3 month of storage condition(temperature) and expired date from bin cared )* | | | | | | |  |  |
| 5. Is you laboratory established inventory management system (observe 3 month data) | | | IFRR and RRF | | | |  |  |
|  |  |  | Bincard | | | |  |  |
|  |  |  | Is FIFO/FEFO applied | | | |  |  |
| 6. Is there installation verification certificate (observe the certificate ) | | | | | | |  |  |
| 6. Is the method verification by your own laboratory *(observe verification log that show the sensitivity and specificity of method )* | | | | | | |  |  |
| 1. Including positive and negative controls periodically *(quality control log sheet)* | | | | | | |  |  |
| 7. Is GeneXpert Machine are calibrated annually with External Run Controls. *(check calibration certificate)* | | | | | | |  |  |
| 8. Total number of module available in this year to give service | | | | | | |  |  |
| 9. Number of Calibration Failed module in this year | | | | | | |  |  |
| 10. Do you have Equipment Failure Contingency Plan | | | | | | |  |  |
| 11. Is sample run in calibration failed module | | | | | | |  |  |
| 12. Number of module Failed after installation | | | | | | |  |  |
| 13. Time of installation (year and month ) | | | | | | |  |  |
| 14. If invalid, no result, or error is obtained, repeating the test with the same specimen. If still invalid, no result, or error, request another specimen for retesting*.(observe one year data from Genexpert machine and registration with its ID and name of patient )* | | | | | | |  |  |
| 15. Description on the reporting of semi-quantitative results (high, medium, low, very low) and Ct (PCR cycle threshold) values*.(observe 3 month registration and reporting form)* | | | | | | |  |  |
| 16. Do you have GeneXpert TAT? (Record their TAT ) | | | | | | |  |  |
| 17. Is Mean Ct values of the Specimen Processing Control and an internal control in the assay are monitored*.(monitoring document)* | | | | | | |  |  |
| 18. Review of all final test results by a second person to minimize reporting errors.( *check the request and reporting form and duty roster )* | | | | | | |  |  |
| 19. Is UPS available for GeneXpert machine | | | | | | |  |  |
| 20. Is UPS the UPS connected to Generator | | | | | | |  |  |
| 21. Do you participate in Proficiency Testing(If yes skip to Q.15) | | | | | | |  |  |
| 22. If no why?*(If no write the reason and skip to Q.35)* | | | | | | |  |  |
| 23. Staff participation in GeneXpert-PT panel testing (observe the duty roster, registration book on the day of TP result report) | | | | | | |  |  |
| 24. Testing EQA panels in the same manner as clinical specimens(observe the duty roster, registration book on the day of TP result report) | | | | | | |  |  |
| 25. If yes where did you get the PT samples? | | | | | IQAS | |  |  |
|  |  |  |  |  | NQAS | |  |  |
|  |  |  |  |  | RQAS | |  |  |
| 26. Did you have an agreement with PT provider? (observer the agreement or registration evidence) | | | | | | |  |  |
| 27. Is the PT provider accredited with ISO/IEC 17043?(observe the certificate if Unknown write UK) | | | | | | |  |  |
| 28. How many times do you receive the PT sample in a year*( One per year, Bi annual, Quarterly) write on the comment section* | | | | | | |  |  |
| 29. When did you start GeneXpert PT participation (*write on the comment section)* | | | | | | |  |  |
| 30. Did you take investigation and action/s for all unacceptable performance? Do you implement corrective action for unacceptable PT performances? (observe the document ) | | | | | | |  |  |
| 31. What problem you face during PT sample processing | Leakage | | | | | |  |  |
|  | PT sample volume | | | | | |  |  |
|  | Procedure instruction | | | | | |  |  |
|  | No problem | | | | | |  |  |
| 33. Challenge of Gene Xpert PT-EQA participation | Commitment of staff | | | | | |  |  |
|  | accessibility of PT samples | | | | | |  |  |
|  | interruption of PT sample | | | | | |  |  |
|  | cost of PT sample | | | | | |  |  |
|  | Donor funding interruption | | | | | |  |  |
|  | Others (specify) | | | | | |  |  |
| 34. challenges during GeneXpert PT sample receiving and reporting | Shipping method | | | | | |  |  |
|  | Delay in transportation*(Estimation of time Delay)* | | | | | |  |  |
|  | Communication gap with provider | | | | | |  |  |
|  | Lack of standard request and reporting paper | | | | | |  |  |
|  | Delays in feedback receiving*(Estimation of time Delay)* | | | | | |  |  |
|  | Feedback missing | | | | | |  |  |
|  | Delay in reporting *(Estimation of time Delay)* | | | | | |  |  |
|  | Other | | | | | |  |  |
| 35. Are you regularly supervised by regional or national TB supervisor | | | | | | National lab |  |  |
|  |  |  |  |  |  | Regional lab |  |  |
|  |  |  |  |  |  | NGO(challenge TB) |  |  |
| 36. If yes ,How many times the supervision done in a year(*1 per year, Biannual, Quarterly )* (*write on the comment section*) | | | | | | National lab |  |  |
|  |  |  |  |  |  | Regional lab |  |  |
|  |  |  |  |  |  | NGO(challenge TB) |  |  |
| 37. Is the supervisors use check list during supervision? | | | | | | |  |  |
| 38. Did you take corrective actions for the identified gaps during supervision? | | | | | | |  |  |
| 39. Do you have improvement plan and implementation records If yes skip to 41 | | | | | | |  |  |
| 40. If no Why?*(If no write the reason and skip to Q.49)* | | | | | | |  |  |
| 41. Monthly review of QC activities by the laboratory supervisor(related to TB) | | | | | | |  |  |
| 42. Documentation of corrective and preventative actions when QC, EQA, QM, and Quality indicator results show unsatisfactory performance occurs | | | | | | |  |  |
| 43. Do you monitor the quality indicators (observe the 3 last month records) | | | | TAT | | |  |  |
|  |  |  |  | IQC | | |  |  |
|  |  |  |  | Customer satisfaction | | |  |  |
|  |  |  |  | EQA | | |  |  |
|  |  |  |  | Equipment down time | | |  |  |
|  |  |  |  | Stock out | | |  |  |
|  |  |  |  | Error rate | | |  |  |

1.5. **Mycobacteria Growth Indicator Tube (MGIT) Culture Checklist**

| **A. Instruction for Relevant Standard Operating Procedures (SOPs)** **.**Indicate “Yes (Y),” “No (N),” or “not Applicable(NA)” for each response below. | | | | | | | | | | |
| --- | --- | --- | --- | --- | --- | --- | --- | --- | --- | --- |
| **SOP No.** | **SOP Title(*Observe and read to see the compliance of SOP with GLI guide line )*** | | | | | **Y** | | **N** | | **NA** |
|  | SOP For Internal quality control of NALC-NaOH and4% Sodium Hydroxide processed sample | | | | |  | |  | |  |
|  | EQA guideline/SOP | | | | |  | |  | |  |
|  | SOP For Method Verification | | | | |  | |  | |  |
|  | SOP For New lot verification | | | | |  | |  | |  |
|  | SOP For Quality Control of Prepared Media, Reagents And Staining Solution | | | | |  | |  | |  |
|  | SOP for Continuous Quality Improvement | | | | |  | |  | |  |
|  | SOP for equipment service maintenance | | | | |  | |  | |  |
|  | SOP for analytical and post analytical | | | | |  | |  | |  |
|  | Standard request and reporting form | | | | |  | |  | |  |
|  | Standard registration log book | | | | |  | |  | |  |
| **B. Instruction for laboratory quality assurance practice assessment** *Indicate “Yes (Y),” “No (N),” or for each response below..* | | | | | | | | | | |
| **Key Elements** | | | | | | | **Y** | | **N** | |
| 1. What type of BSC do you used for sample processing? | | | | | | |  | |  | |
| 2. Does your BSC calibrate regularly?*(Observe 2 year certificate of BSC)* | | | | | | |  | |  | |
| 3. If No for how long it takes for recalibration *(write on the comment section)* | | | | | | |  | |  | |
| 4. Does your negative pressure calibrate regularly ?*(Observe 2 year certificate of BSC)* | | | | | | |  | |  | |
| 5. If No for how long it takes for recalibration*(write on the comment section)* | | | | | | |  | |  | |
| 6. Inoculating a negative (end and start )control in an MGIT tube to check for sterility of the processing reagents and monitor for cross-contamination.(*check quality control log and MGIT result print out )* | | | | | | |  | |  | |
| 7. Inoculating a positive sputum processing control (suspended pellet) in an MGIT tube to monitor NaOH killing of MTB.( *check 3 month quality control log and MGIT result print out )* | | | | | | |  | |  | |
| 8. Prior to putting new lot numbers of media(MGT tube), Growth Supplement, and PANTA into use, checking the performance by culturing*.( by taking one result and back to MGIT result print out also observe at least 3 lot of reagent in lot to lot verification log book to know the continuity)* | | | | | | |  | |  | |
| 9. Checking the storage conditions and expiration date of the lyophilized PANTA (maintain at 2-8°C*).( check 3 month of the storage condition and expired date from Bincard )* | | | | | | |  | |  | |
| 10. Storing reconstituted PANTA at 2-8°C and using it within 5 days. Do not freeze PANTA*).( check the storage condition )* | | | | | | |  | |  | |
| 11. Not using expired and not properly stored MGIT reagent *.(check 3 month of storage condition (temperature) and expired date from bin card )* | | | | | | |  | |  | |
| 12. testing each new lot/batch of in-house prepared media for sterility, growth performance | | | NALC-NaOH, | | | |  | |  | |
|  |  |  | 4%NaOH | | | |  | |  | |
|  |  |  | BHI/blood agar | | | |  | |  | |
|  |  |  | PBS buffer*(including PH)* | | | |  | |  | |
|  |  |  | Albumin | | | |  | |  | |
| 13. Performing daily/monthly MGIT instrument maintenance.( observe 3 month daily/monthly maintenance) | | | | | | |  | |  | |
| 14. Annual MGIT machine calibration(check the calibrator of MGIT Machine | | | | | | |  | |  | |
| 15. Is there installation verification certificate *(observe the certificate )* | | | | | | |  | |  | |
| 16. Is the MGIT machine method verified by your own laboratory (observe verification document ) | | | | | | |  | |  | |
| 17. Labeling the MGIT tube with patient identification, specimen number, and date of inoculation*.(observe inoculated MIGT tube)* | | | | | | |  | |  | |
| 18. Is positive result loaded within 0 day when the instrument-generated time to detection (TTD) in days and hours.(observe 3 month MIGT print out) | | | | | | |  | |  | |
| 19. Preparing a blood agar plate (BAP) for all positive cultures to rule out contamination *(observe 3 month of work sheet*). | | | | | | |  | |  | |
| 20. Inspecting all negative cultures for growth prior to discarding*.(see 3 month MGIT negative result print out* ) | | | | | | |  | |  | |
| 21. Review of all final test results by a second person to minimize reporting errors.( *check 3 month the request and reporting form and duty roster )* | | | | | | |  | |  | |
| 22. Write MGIT TAT of laboratory*?* | | | | | | |  | |  | |
| 23. Is UPS avail for MGT machine | | | | | | |  | |  | |
| 24. Is UPS the UPS connected to Generator | | | | | | |  | |  | |
| 25. Comparing of results to the corresponding results on worksheets/LIS/registers (laboratory source documents) to ensure accuracy before reporting. *(take one MGIT result follow the process from sample reception to result delivered)* | | | | | | |  | |  | |
| 26. Do you participate in Proficiency Testing(If yes skip to Q.28) | | | | | | |  | |  | |
| 27. If no why?*(If no write the reason and skip to Q.40)* | | | | | | |  | |  | |
| 28. If yes where did you get the PT samples? | | | | | IQAS | |  | |  | |
|  |  |  |  |  | NQAS | |  | |  | |
|  |  |  |  |  | RQAS | |  | |  | |
| 29. Staff participation in MGIT-PT panel testing (observe the duty roster, registration book on the day of TP result report) | | | | | | |  | |  | |
| 30. Testing EQA panels in the same manner as clinical specimens(observe the duty roster, registration book on the day of TP result report) | | | | | | |  | |  | |
| 31. Did you have an agreement with PT provider? (observer the agreement or registration evidence) | | | | | | |  | |  | |
| 32. Is the PT provider accredited with ISO/IEC 17043?(observe the certificate if Unknown write UK) | | | | | | |  | |  | |
| 33. How many times do you receive the PT sample in a year*( One per year, Bi annual, Quarterly) write on the comment section* | | | | | | |  | |  | |
| 34. When did you start TB PT participation | | | | | | |  | |  | |
| 35. What was your past culture PT performance with (2016-2018 if available?) write on the comment section | | | | | | |  | |  | |
| 36. Did you take investigation and action/s for all unacceptable performance? (observe the document ) | | | | | | |  | |  | |
| 37. What problem you face during PT sample processing | | Leakage | | | | |  | |  | |
|  |  | PT sample volume | | | | |  | |  | |
|  |  | Procedure instruction | | | | |  | |  | |
|  |  | No problem | | | | |  | |  | |
| 38. Challenge of PT-EQA participation | | Commitment of staff | | | | |  | |  | |
|  |  | accessibility of PT samples | | | | |  | |  | |
|  |  | interruption of PT sample | | | | |  | |  | |
|  |  | cost of PT sample | | | | |  | |  | |
|  |  | Donor funding interruption | | | | |  | |  | |
|  |  | Others (specify) | | | | |  | |  | |
| 39. challenges during PT sample receiving and reporting?(can be select more than one) | | Shipping method | | | | |  | |  | |
|  |  | Delay in transportation *(Estimation of time Delay)* | | | | |  | |  | |
|  |  | Communication gap with provider | | | | |  | |  | |
|  |  | Lack of standard request and reporting paper | | | | |  | |  | |
|  |  | Delays in feedback receiving *(Estimation of time Delay)* | | | | |  | |  | |
|  |  | Feedback missing | | | | |  | |  | |
|  |  | Delay in reporting *(Estimation of time Delay)* | | | | |  | |  | |
|  |  | Other | | | | |  | |  | |
| 40. Are you regularly supervised by regional or national TB supervisor if yes skip to 42 | | | | National lab | | |  | |  | |
|  |  |  |  | Regional lab | | |  | |  | |
|  |  |  |  | NGO(challenge TB) | | |  | |  | |
| 41. If no why?(If no write the reason and skip to Q.45) | | | | | | |  | |  | |
| 42. If yes ,How many times the supervision done in a year(*1 per year, Biannual, Quarterly )* (*write on the comment section*) | | | | | National lab | |  | |  | |
|  |  |  |  |  | Regional lab | |  | |  | |
|  |  |  |  |  | NGO(challenge TB) | |  | |  | |

1.6. **Solid Media Culture Checklist**

| A. **Instruction for Relevant Standard Operating Procedures (SOPs)** .Indicate “Yes (Y),” “No (N),” or “not Applicable (NA)” for each response below. Justify or explain all “N” or “NA” responses in the “Comments” column*.* | | | | | |
| --- | --- | --- | --- | --- | --- |
| **SOP No.** | **SOP Title(*Observe and read to see the compliance of SOP with GLI guide line )*** | **Y** | **N** | **NA** | **Comment** |
|  | SOP For Internal quality control of NALC-NaOH and 4% Sodium Hydroxide processed sample |  |  |  |  |
|  | EQA guideline/SOP |  |  |  |  |
|  | SOP For Method Verification |  |  |  |  |
|  | SOP For New lot verification |  |  |  |  |
|  | SOP For Quality Control of Prepared Media, Reagents And Staining Solution |  |  |  |  |
|  | SOP for Continuous Quality Improvement |  |  |  |  |
|  | SOP for equipment service maintenance |  |  |  |  |
|  | SOP for analytical and post analytical |  |  |  |  |
|  | Standard request and reporting form |  |  |  |  |
|  | Standard registration log book |  |  |  |  |

| B. **Instruction for laboratory quality assurance practice assessment** *.Indicate “Yes (Y),” “No (N),” or “Partial practice or luck of continuity (P)” for each response below. Justify or explain all “N” or “P” responses in the “Comments” column.* | | | | | | | | | | |
| --- | --- | --- | --- | --- | --- | --- | --- | --- | --- | --- |
| **Key Elements** | | | | | | | **Y** | **N** | **P** | **If”no and partial” write write the reason why not perform** |
| 1. What type of BSC does you for sample processing? | | | | | | |  |  |  |  |
| 2. Does your BSC calibrate regularly?*(Observe 2 year certificate of BSC)* | | | | | | |  |  |  |  |
| 3. If No for how long it takes for recalibration *(write on the comment section)* | | | | | | |  |  |  |  |
| 4. Does your negative pressure calibrate regularly ?*(Observe 2 year certificate of BSC)* | | | | | | |  |  |  |  |
| 5. If No for how long it takes for recalibration*(write on the comment section)* | | | | | | |  |  |  |  |
| 6. Inoculating a negative (start and End)on a solid medium to check for sterility of the processing reagents and monitor for cross-contamination.( *check quality control log and current inoculated LJ )* | | | | | | |  |  |  |  |
| 7. Inoculating a positive sputum processing control (resuspended pellet) on a solid medium to monitor NaOH killing of MTB.(*check quality control log and current inoculated LJ )* | | | | | | |  |  |  |  |
| 8. Testing each new lot/batch of in-house prepared media for sterility, growth performance. | | NALC-NaOH, | | | | |  |  |  |  |
|  |  | 4%NaOH | | | | |  |  |  |  |
|  |  | BHI/blood agar | | | | |  |  |  |  |
|  |  | PBS buffer*(including PH)* | | | | |  |  |  |  |
|  |  | Albumin | | | | |  |  |  |  |
|  |  | LJ media | | | | |  |  |  |  |
| 9. Routine maintenance and cleaning of the incubators | | | | | | |  |  |  |  |
| 10. Temperature Calibration for incubators(certificate of thermometer ) | | | | | | |  |  |  |  |
| 11. Not using expired and not properly stored media.(check the expired date on prepared media and storage condition ) | | | | | | |  |  |  |  |
| 12. Recording growth results weekly on the laboratory worksheet.(compare 3 month of the reported result with work sheet) | | | | | | |  |  |  |  |
| 13. Incubating solid media for reporting a negative result for 8 weeks .(*check 3 month of date of inoculation to report on work sheet )* | | | | | | |  |  |  |  |
| 14. Recording growth at the final reading interval according to the WHO/IUALTD) Standardized reporting scheme. *(check 3 month data on final registration book and work sheet)* | | | | | | |  |  |  |  |
| 15. Review of all final test results by a second person to minimize reporting errors*(check 3 month data on the request and reporting form and duty roster )* | | | | | | |  |  |  |  |
| 16. Write LJ result report TAT of laboratory? | | | | | | |  |  |  |  |
| 17. Is UPS avail for incubator | | | | | | |  |  |  |  |
| 18. Comparing of results to the corresponding results on worksheets/LIS/registers (laboratory source documents) to ensure accuracy before reporting. *( take one LJ result follow the process from sample reception to result delivered )* | | | | | | |  |  |  |  |
| 19. Do you participate in Proficiency Testing(If yes skip to Q.21) | | | | | | |  |  |  |  |
| 20. If no why?*(If no write the reason and skip to Q.33)* | | | | | | |  |  |  |  |
| 21. If yes where did you get the PT samples? | | | | IQAS | | |  |  |  |  |
|  |  |  |  | NQAS | | |  |  |  |  |
|  |  |  |  | RQAS | | |  |  |  |  |
| 22. Did you have an agreement with PT provider? (observer the agreement or registration evidence) | | | | | | |  |  |  |  |
| 23. Is the PT provider accredited with ISO/IEC 17043?(observe the certificate if Unknown write UK) | | | | | | |  |  |  |  |
| 24. How many times do you receive the PT sample in a year( One per year, Bi annual, Quarterly) *write on the comment section* | | | | | | |  |  |  |  |
| 25. Staff participation in LJ-PT panel testing (observe the duty roster, registration book on the day of TP result report) | | | | | | |  |  |  |  |
| 26. Testing EQA panels in the same manner as clinical specimens(observe the duty roster, registration book on the day of TP result report) | | | | | | |  |  |  |  |
| 27. When did you start PT participation *write on the comment section* | | | | | | |  |  |  |  |
| 28. What problem you face during PT sample processing | Leakage | | | | |  | |  |  |  |
|  | PT sample volume | | | | |  | |  |  |  |
|  | Procedure instruction | | | | |  | |  |  |  |
|  | No problem | | | | |  | |  |  |  |
| 29. Challenge of PT-EQA participation | Commitment of staff | | | | |  | |  |  |  |
|  | accessibility of PT samples | | | | |  | |  |  |  |
|  | interruption of PT sample | | | | |  | |  |  |  |
|  | cost of PT sample | | | | |  | |  |  |  |
|  | Donor funding interruption | | | | |  | |  |  |  |
|  | Others (specify) | | | | |  | |  |  |  |
| 30. Challenges during PT sample receiving and reporting? | Shipping method | | | | |  | |  |  |  |
|  | Delay in transportation *(Estimation of time Delay)* | | | | |  | |  |  |  |
|  | Communication gap with provider | | | | |  | |  |  |  |
|  | Lack of standard request and reporting paper | | | | |  | |  |  |  |
|  | Delays in feedback receiving *(Estimation of time Delay)* | | | | |  | |  |  |  |
|  | Feedback missing | | | | |  | |  |  |  |
|  | Delay in reporting *(Estimation of time Delay)* | | | | |  | |  |  |  |
|  | Other | | | | |  | |  |  |  |
| 33. Are you regularly supervised by regional or national TB supervisor | | | National lab | | |  | |  |  |  |
|  |  |  | Regional lab | | |  | |  |  |  |
|  |  |  | NGO(challenge TB) | | |  | |  |  |  |
| 34. If no why?(If no write the reason and skip to Q.38) | | | | | |  | |  |  |  |
| 35. If yes ,How many times the supervision done in a year(*1 per year, Biannual, Quarterly )* (*write on the comment section*) | | | | | National lab |  | |  |  |  |
|  |  |  |  |  | Regional lab |  | |  |  |  |
|  |  |  |  |  | NGO(challenge TB) |  | |  |  |  |

1.7. **Lateral Flow Assays for the rapid ID of MTBC**

| **A. Instruction for Relevant Standard Operating Procedures (SOPs)** .Indicate “Yes (Y),” “No (N),” or “not Applicable (NA)” for each response below. | | | | | |
| --- | --- | --- | --- | --- | --- |
| **SOP No.** | **SOP Title(*Observe and read to see the compliance of SOP with GLI guide line )*** | **Y** | **N** | **NA** | **Comment** |
|  | EQA guideline/SOP |  |  |  |  |
|  | SOP For Method Verification |  |  |  |  |
|  | SOP For New lot verification |  |  |  |  |
|  | SOP for analytical and post analytical |  |  |  |  |
|  | Standard registration |  |  |  |  |

| B. **Instruction for laboratory quality assurance practice assessment** *.Indicate “Yes (Y),” “No (N),” for each response below.* | | |
| --- | --- | --- |
| **Key Elements** | **Y** | **N** |
| 2. Do you Confirm the presence of MTB vs. non-MTB at each culture is AFB positive.(*observe 3month data on work sheet and registration )* |  |  |
| 3. Including positive and negative controls periodically *(observe 3 month data on quality control log sheet)* |  |  |
| 4. What type of BSC do you used for Identification? |  |  |
| 5. Does your BSC calibrate regularly?*(Observe 2 year certificate of BSC)* |  |  |
| 6. Performing an MPT64 antigen test only on cultures confirmed as being AFB positive and BHI negative. (Observe 3 month data on work sheet |  |  |
| 7. Not using expired and not properly stored media.(check the expired date on prepared media and storage condition ) |  |  |
| 8. Testing the MGIT culture within 2-5 days of the instrument signaling positive.(observe 3 month data on MIGIT result print out date of positivity of MGIT and result report) |  |  |
| 9. Is the method verified by your own laboratory (observe the document) |  |  |
| 10. Re-incubating and retesting all BHI negative and Smear negative (observe 3 month of data on work sheet ) |  |  |
| 11. Testing new lot numbers with negative and positive controls before putting the MPT64 antigen kits into use. (observe 3 batch lot to lot verification log ) |  |  |
| 12. Do you used rapid antigen test for culture PT panel identification |  |  |

.8. **Hain Line Probe Assays Checklist**

| **A. Instruction for Relevant Standard Operating Procedures (SOPs)** Indicate “Yes (Y),” “No (N),” or “not Applicable(NA)” for each response below. | | | | |  | |
| --- | --- | --- | --- | --- | --- | --- |
| **SOP No.** | **SOP Title(*Observe and read to see the compliance of SOP with GLI guide line )*** | **Y** | **N** | **Comment** | |  |
|  | EQA guideline/SOP |  |  |  | |  |
|  | SOP For Method Verification |  |  |  | |  |
|  | SOP For New lot verification |  |  |  | |  |
|  | SOP For Quality Control of LPA |  |  |  | |  |
|  | SOP for Continuous Quality Improvement |  |  |  | |  |
|  | SOP for equipment service maintenance |  |  |  | |  |
|  | Standard request and reporting form |  |  |  | |  |
|  | Standard registration log book |  |  |  | |  |

| **B.Instruction for laboratory quality assurance practice assessment**: *Indicate “Yes (Y),” “No (N),” for each response below.* | | | | | | | | |
| --- | --- | --- | --- | --- | --- | --- | --- | --- |
| **Key Elements** | | | | | | **Y** | **N** | **NA** |
| 1. Including positive and negative controls with each batch of specimens/cultures or one batch per week*.(observe one year quality control log and each batch of strip )* | | | | | |  |  |  |
| 2. What type of BSC used for DNA extraction procedure | | | | | |  |  |  |
| 3. Does your BSC calibrate regularly?*(Observe 2 year certificate of BSC)* | | | | | |  |  |  |
| 4. Areas for master-mix preparation, DNA template addition/PCR, and detection are physically separated.(observe the work Area ) | | | | | |  |  |  |
| 5. For the MTBDR*plus* assay, MTB must be detected for INH and RIF results to be valid.(observe one year MTBDR*pluss stripe* ) | | | Amplification control | | |  |  |  |
|  |  |  | Conjugate control | | |  |  |  |
|  |  |  | TUB | | |  |  |  |
|  |  |  | Locus control | | |  |  |  |
| 6. For the MTBDR*sl* assay, MTB must be detected for fluoroquinolone and aminoglycoside/cyclic peptide results to be valid.(*observe 3 month MTBDRpluss stripe )* | | | Amplification control | | |  |  |  |
|  |  |  | Conjugate control | | |  |  |  |
|  |  |  | TUB | | |  |  |  |
|  |  |  | Locus control | | |  |  |  |
| 7. For the MTBDR*plus* and MTBDR*sl* assays, if invalid results are obtained, repeating with newly extracted DNA from the same specimen. If still invalid, request another specimen (track by name and ID). | | | | | |  |  |  |
| 8. Write LPA TAT of laboratory? | | From sediment | | | |  |  |  |
|  |  | From culture | | | |  |  |  |
| 9. QC testing new lots of LPA kits with appropriate controls before using.*(check one year data of lot to lot verification log with LPA strip)* | | | | | |  |  |  |
| 10. Not using expired and not properly stored media.(check the expired date on prepared media and storage condition ) | | | | | |  |  |  |
| 11. Routine maintenance and cleaning of *(3 month routine maintenance* ) | | | | Heatblock, | |  |  |  |
|  |  |  |  | Thermal Cycler | |  |  |  |
|  |  |  |  | PCR hood | |  |  |  |
|  |  |  |  | TwinCubator | |  |  |  |
|  |  |  |  | Water bath | |  |  |  |
|  |  |  |  | Micro centrifuge(speed and time) | |  |  |  |
| 14. Calibration of Automatic pipette | | | | | |  |  |  |
| 15. Review of all final test results by a second person to minimize reporting errors.(Check 3 month of data from duty roster and reporting form) | | | | | |  |  |  |
| 16. Is UPS available for twincubetor | | | | | |  |  |  |
| 17. Is UPS the UPS connected to Generator | | | | | |  |  |  |
| 18. Do you participate in Proficiency Testing(If yes skip to Q.20) | | | | | |  |  |  |
| 19. If no why?*(If no write the reason and skip to Q.31* | | | | | |  |  |  |
| 20. If yes where did you get the PT samples? | | | | | IQAS |  |  |  |
|  |  |  |  |  | NQAS |  |  |  |
|  |  |  |  |  | RQAS |  |  |  |
| 20. Did you have an agreement with PT provider? (observer the agreement or registration evidence) | | | | | |  |  |  |
| 21. Is the PT provider accredited with ISO/IEC 17043?(observe the certificate if Unknown write UK) | | | | | |  |  |  |
| 22. How many times do you receive the PT sample in a year( One per year, Bi annual, Quarterly) *write on the comment section* | | | | | |  |  |  |
| 23. Staff participation in LPA-PT panel testing (observe the duty roster, registration book on the day of TP result report) | | | | | |  |  |  |
| 24. Testing EQA panels in the same manner as clinical specimens(observe the duty roster, registration book on the day of TP result report) | | | | | |  |  |  |
| 25. When did you start TB PT participation | | | | | |  |  |  |
| 26. What problem you face during PT sample processing | Leakage | | | | |  |  |  |
|  | PT sample volume | | | | |  |  |  |
|  | Procedure instruction | | | | |  |  |  |
|  | No problem | | | | |  |  |  |
| 27. Challenge of PT-EQA participation | Commitment of staff | | | | |  |  |  |
|  | accessibility of PT samples | | | | |  |  |  |
|  | interruption of PT sample | | | | |  |  |  |
|  | cost of PT sample | | | | |  |  |  |
|  | Donor funding interruption | | | | |  |  |  |
|  | Others (specify) | | | | |  |  |  |
| 28. Challenges during PT sample receiving and reporting? | Shipping method | | | | |  |  |  |
|  | Delay in transportation *(Estimation of time Delay)* | | | | |  |  |  |
|  | Communication gap with provider | | | | |  |  |  |
|  | Lack of standard request and reporting paper | | | | |  |  |  |
|  | Delays in feedback receiving *(Estimation of time Delay)* | | | | |  |  |  |
|  | Feedback missing | | | | |  |  |  |
|  | Delay in reporting *(Estimation of time Delay)* | | | | |  |  |  |
|  | Other | | | | |  |  |  |

January 18, 2022

Yours, Sincerely

Yeshiwork Abebaw
