# Supplementary material for: Quality assurance practices in tuberculosis diagnostic health facilities in Ethiopia
Source: PLoS One. 2022 Jun 9;17(6):e0269601. doi: 10.1371/journal.pone.0269601 (PMC9182608; doi:10.1371/journal.pone.0269601)
Supplement: Attachment — Submitted filename: Response to Reviewers.docx [file pone.0269601.s004.docx]

**PLOS ONE FEEDBACK APRIL 2022**

**QUALITY ASSURANCE PRACTICES IN TUBERCULOSIS DIAGNOSTIC HEALTH FACILITIES IN ETHIOPIA**

Dear Editor,

Thank you for your informative comments and thoughtful comments on our manuscript. We have addressed your and the reviewer comments one by one. We also appreciate the reviewers for their critical observation and informative comments which radically improved our manuscript quality. We have provided point-by-point response to the reviewer 2 comments below with table. Also, we would like to inform you that we have used hyperlink to indicate where we made changes in the previous version of the manuscript based on the reviewers’ comments.

Responses to Editorial manager

| No | Editorial manager comment | Responses to Editorial manager |
| --- | --- | --- |
| 1 | Line 95. “Blinded rechecking of TB diagnostic centers”. Is this blinded rechecking of TB slides? | Thank you very much for your critical observation. Yes, we need to say blinded rechecking of TB slides. We apologize for the error and have corrected it in line 106 |
| 2 | Line 174. “Date” should be replace by “data”. In addition, there is need for minor edits and in general the text needs to be streamlined so the language can flow. I have given a few examples below, but there are several more instances in the manuscript. | Thank you very much for your insightful observation, we apologize for making confusion. We rephrase the word in line 186. Yes, there were number of grammar error. We have corrected accordingly as observed in the track change of the general the text. |
| 3 | Line 75. Should read: “…quality assurance of testing…” | Thank you very much for your insightful comments, We rephrased the word in line 80 |
| 4 | Lines 79-80. This sentence remains unclear. Suggestions in italics: “Quality control ensures that all operations in the TB diagnostic laboratory are monitored, including equipment checks and checking new lots of staining solutions | Thank you very much for your critical observation and we apologize for making confusion, We rephrased the word and corrected in line 86 |
| 5 | Line 81. “EQA plays a vital role in monitoring laboratory performance…”  Line 82. ”… quality services.” | Thank you very much for your observation. We removed the sentence in line 90 and rephrase the word in line 89 |
|  | Lines 83-84. “One of the End TB strategic goals in 2020 was to enroll all TB diagnostic facilities in EQA”. | Thank you very much for your observation. We rephrased the word and corrected in line 93 |
|  | Line 89. “…measurable laboratory improvement in a resource limited settings”. | Thank you very much for your observation. We rephrased the word and corrected in line 90 |
|  | Line 91. “Ethiopia National TB prevention and control program with…”. If this is the title of a program, the relevant words should start with capital letters. | Thank you very much for your observation. We corrected in line 102 |

**Sincerely**

**Yeshiwork Abebaw**
